# Van der Waals Epitaxy of 2D Gallium Telluride on Graphene: Growth Dynamics and Principal Component Analysis

**DOI:** 10.1002/smll.202503993

**Published:** 2025-05-02

**Authors:** Michele Bissolo, Michael Hanke, Raffaella Calarco, Jonathan J. Finley, Gregor Koblmüller, J. Marcelo J. Lopes, Eugenio Zallo

**Affiliations:** ^1^ Walter‐Schottky‐Institut and TUM School of Natural Sciences Technische Universität München Am Coulombwall 4 85748 Garching Germany; ^2^ Paul‐Drude‐Institut für Festkörperelektronik Leibniz‐Institut im Forschungsverbund Berlin e.V. Hausvogteiplatz 5‐7 10117 Berlin Germany; ^3^ Institute for Microelectronics and Microsystems (IMM) Consiglio Nazionale delle Ricerche (CNR) Via del Fosso del Cavaliere 100 Rome 00133 Italy; ^4^ Institute of Solid State Physics Technical University Berlin Hardenbergstrasse 36 10623 Berlin Germany

**Keywords:** 2D materials, gallium telluride, heterostructure, machine learning, van der waals epitaxy

## Abstract

A scalable epitaxy of 2D layered materials and heterostructures constitutes a crucial step in developing novel optoelectronic applications based on high‐crystalline quality 2D materials. Here, the formation of continuous, strain‐free, high‐crystalline quality 2D hexagonal gallium telluride (h‐GaTe) directly on epitaxial graphene using molecular beam epitaxy is demonstrated. Morphological and structural characterizations evidence a coherent layer at the heterostructure interface having an in‐plane lattice constant of 4.05 ± 0.01 $\dot{A}$. The few‐layer thick graphene determines the epitaxial registry of the h‐GaTe with grains of sixfold symmetry and a multilayer‐type homoepitaxial growth. Deposition temperature‐ and time‐dependent surface topography indicate that the interlayer diffusion of adatoms plays a crucial role in achieving smooth GaTe films. Contrastive principal component analysis allows for screening large in situ diffraction data as a function of growth parameters. In this way, the trajectory of the 2D h‐GaTe growth is mapped through phase space. These results are relevant for integrating epitaxial material in the fabrication of high‐performance multifunctional devices.

## Graphical abstract

Van der Waals (vdW) epitaxy of continuous, strain‐free, high‐crystalline quality vdW heterostructure consisting of 2D hexagonal gallium telluride (h‐GaTe) on epitaxial graphene on SiC. The interlayer diffusion of adatoms plays a crucial role in achieving smooth GaTe films.

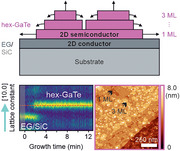

## 1 Introduction

The creation of van der Waals heterostructures (vdWH)^[^ ^1^, ^2^ ^]^ by stacking combinations of 2D crystals allows for the design of novel devices with highly emergent functionalities.^[^ ^3^, ^4^ ^]^ Specific examples include harnessing coupled ferroelectric and superconductive states,^[^ ^5^ ^]^ electrically tunable Nernst effect,^[^ ^6^ ^]^ and interlayer excitons.^[^ ^7^ ^]^ Post transition metal chalcogenides (PTMCs) have sparked great interest among the family of 2D materials beyond graphene due to their unique electronic^[^ ^8^, ^9^ ^]^ and optoelectronic^[^ ^10^, ^11^, ^12^ ^]^ properties. PTMC gallium telluride in its hexagonal phase (h‐GaTe) is a layered semiconductor belonging to the P6_3_/mmc space group,^[^ ^13^ ^]^ with predicted half metallicity due to the almost flat valence band in the few‐layer limit.^[^ ^14^ ^]^ While thermodynamically metastable, this phase shows capability for high‐temperature thermoelectrics^[^ ^15^ ^]^ and improved optoelectronic performances when combined with graphene.^[^ ^16^ ^]^ Most atomically thin 2D‐layered materials are obtained through mechanical exfoliation of monolayers (MLs) from bulk crystals and can be stacked to form vdWHs. Moreover, the scalability of this process and the risk of interlayer contamination remain a concern despite significant advancements in interface quality achieved through novel assembly techniques.^[^ ^17^, ^18^, ^19^ ^]^ In addition, information of the pristine PTMC is hidden by its surface reactivity resulting in modified device performances and occluded physical findings.^[^ ^20^ ^]^ By using molecular beam epitaxy (MBE) to synthesize layered materials, new insights into the process of domain formation can be obtained, revealing vdWH with sharp interfaces.^[^ ^21^ ^]^ Interestingly, lower nucleation densities typically stem from vdW epitaxy (2D material on 2D substrate) as compared to (quasi)‐vdW epitaxy (2D material on 3D substrate).^[^ ^22^, ^23^ ^]^ However, which growth regime leads to specific island morphology remains unclear. Recently, h‐GaTe has been epitaxially synthesized on passivated silicon but some defects at the interface produced regions with local strain^[^ ^24^ ^]^ that can act as current leakage path for electronic devices. In this work, we demonstrate vdW‐type MBE of h‐GaTe on epitaxial graphene with thicknesses ranging from 3 to 27 MLs. Electron and X‐ray diffraction measurements confirm the absence of strain relaxation in the GaTe, which is epitaxially aligned with the epitaxial graphene (EG) and forms a GaTe/EG vdWH of preserved structural quality, while the interaction with the underlying SiC substrate potentially yields a second in‐plane orientation. Morphological analysis shows that multilayer growth is favored after the first coherent layer is deposited on EG due to reduced interlayer adatom mobility during homoepitaxy. Kinematic models support this observation by showing that the growth dynamics do not follow a pure layer‐by‐layer mode. Finally, the in‐situ electron diffraction data collected across a range of substrate temperatures and growth times are analyzed using contrastive principal component analysis (cPCA) to identify and classify distinct regimes of the growth of 2D h‐GaTe. This study underlines the role of the epitaxial registry and kinetics for the realization of high‐quality vdWH and demonstrate a potential vdWH candidate for novel thermoelectric and optoelectronic devices.

## 2 Results and Discussion

**Figure 1:**
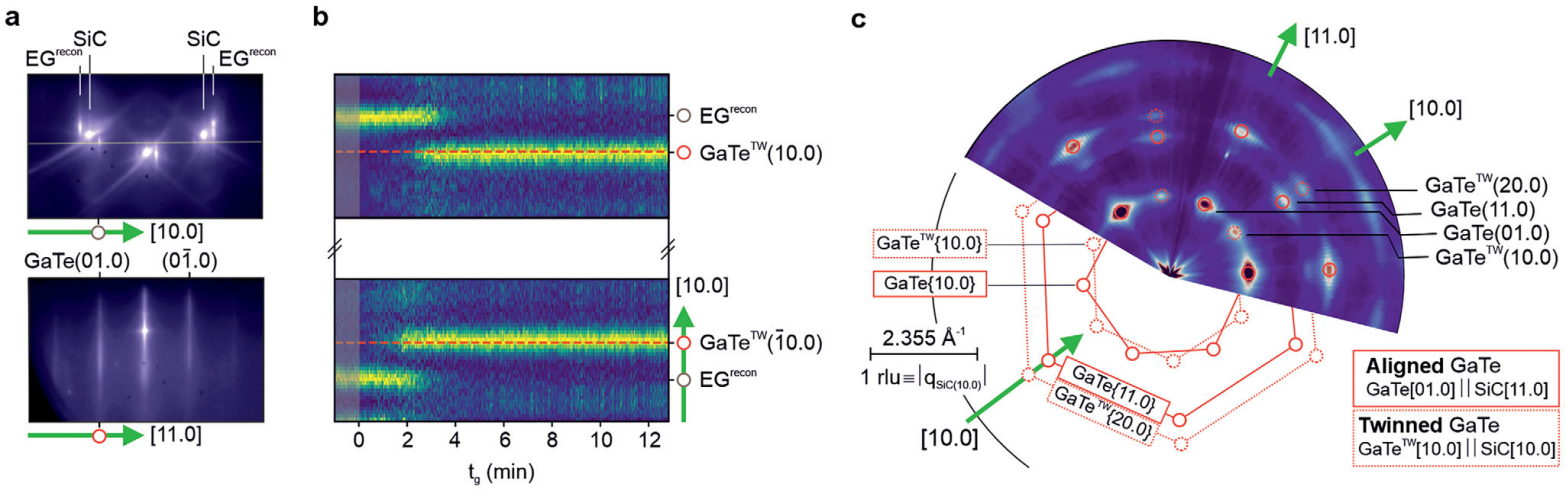
Formation of van der Waals heterostructure observed via in‐situ diffraction. Static reflection high‐energy electron diffraction (RHEED) patterns of (a, top) pure epitaxial graphene (EG) layer grown on 4H‐SiC(00.1) substrate and (a, bottom) few‐layers GaTe overgrown by molecular beam epitaxy (MBE) on EG/SiC. The patterns are obtained by taking images perpendicular to the [10.0] and [11.0] directions of the SiC substrate, respectively, at a substrate temperature (*T_sub_*) of 30 °C, as highlighted by the bold green arrows at the outset of each image. The superstructure observed in (a, top) in proximity to both mirror‐symmetric EG reflections is the 6($\sqrt{3}\times \sqrt{3}$)R30° surface reconstruction (EG^recon^). b) Time‐dependent linear RHEED scans during the initial stages of the MBE growth, recorded at the same position as the horizontal line in (a, top). The dashed red lines indicate the GaTe^TW^{10.0} positions at the end of the growth and confirm the lack of strain relaxation during the whole deposition time (*t*_*g*_). c) In‐plane RHEED map derived from three monolayers (MLs) of GaTe that encompasses a multifaceted array of data. The contributions from GaTe and a twinned GaTe^TW^ have been indexed.

Our vdWH consists of vertically stacked 2D GaTe deposited by MBE on EG layers on (c‐plane) 4H‐SiC(00.1) substrates. Regarding the EG/SiC substrate, the large SiC terraces are covered with ML graphene, whereas few‐layer graphene (mainly bilayer and trilayer growth) is present at the step edges. Further details are provided in the Experimental Section and Figure S1 (Supporting Information). In **Figure** **1**a we present static RHEED patterns for an equivalent section of reciprocal space as spanned by the [10.0] and [11.0] (in‐plane) vectors, respectively, and the [00.1] (out‐of‐plane) surface normal. Please note that we use the four‐component Miller‐Bravais notation for hexagonal symmetry, i.e., (hklm) = (h k ‐(h+k) m) = (hk.m).

The RHEED pattern in Figure 1a (top) corresponds to the initial surface with pure EG grown onto the SiC substrate. The superstructure observed in proximity to both mirror‐symmetric SiC reflections can be attributed to the 6($\sqrt{3}\times \sqrt{3}$)R30° surface reconstruction from the EG/SiC interface layer (EG^recon^).^[^ ^25^ ^]^ The pattern undergoes a significant transformation after depositing 27 MLs of GaTe (see bottom Figure 1a and Experimental Section), as evidenced at a substrate temperature (*T*_*sub*_) of 30 °C: The EG signal disappears and the reflections from GaTe{01.0} emerge.

Figure 1b provides dynamic RHEED information. Individual line scans, as marked by the faint horizontal line in Figure 1a (top), i.e., running along [10.0], are normalized, stacked, and plotted as a function of growth time (*t*_*g*_) with a frame rate of 157 ms. Thereby, we can track different stages in situ. Prior to the deposition of GaTe and up to a (deposition) time of approximately 2 min, only the reflections due to EG^recon^ are visible. Afterward, the EG^recon^ signal disappears, and features arising from the GaTe layer appear at smaller reciprocal vector **q**_[10.0]_ and become the dominant signal (Figure S2, Supporting Information). From the final RHEED line scan (taken at the end of the growth) we determine a GaTe in‐plane lattice parameter of 4.05 ± 0.02 $\dot{A}$. This value perfectly matches the one obtained for the only report on bulk h‐GaTe.^[^ ^26^ ^]^ In contrast with the case of GaTe on silicon,^[^ ^24^ ^]^ where a decrease of the lattice parameter at the very beginning of the growth and a resulting 1% tensile strain for 11 MLs were reported, the nominal interface between GaTe and EG is well preserved and the 2D GaTe layer remains strain‐free, even in the limit of thicker films of up to 27 MLs. This is confirmed by the dashed red lines in Figure 1b that correspond to the position of the (10.0) peak at the end of the growth run, matching the value during the first minutes of deposition. The unstrained in‐plane lattice parameter provides compelling evidence for the vdW‐type of epitaxy resulting in ideal heterostructures, as also reported for 2D magnets grown on the same type of graphene templates.^[^ ^27^, ^28^ ^]^ For more information on the in‐plane registry and the crystal symmetry of the epitaxial layer, we have taken an in‐situ azimuthal RHEED of 3 MLs 2D GaTe, as shown in Figure 1c. The pole map consists of the static RHEED patterns recorded parallel to the surface, namely the respective planes are defined by the (in‐plane) vectors [10.0] and [11.0], where we use SiC as reference. The sharp diffraction peaks manifest sixfold symmetry (GaTe{10.0}) and weaker diffracted intensities rotated by 30° around the main maxima (GaTe^TW^{10.0}). This demonstrates the formation of 30° rotational domains as a result of the vdW epitaxy.^[^ ^29^ ^]^ Corresponding families of reflections are indicated as straight (aligned) and dashed (twinned) hexagons.

**Figure 2:**
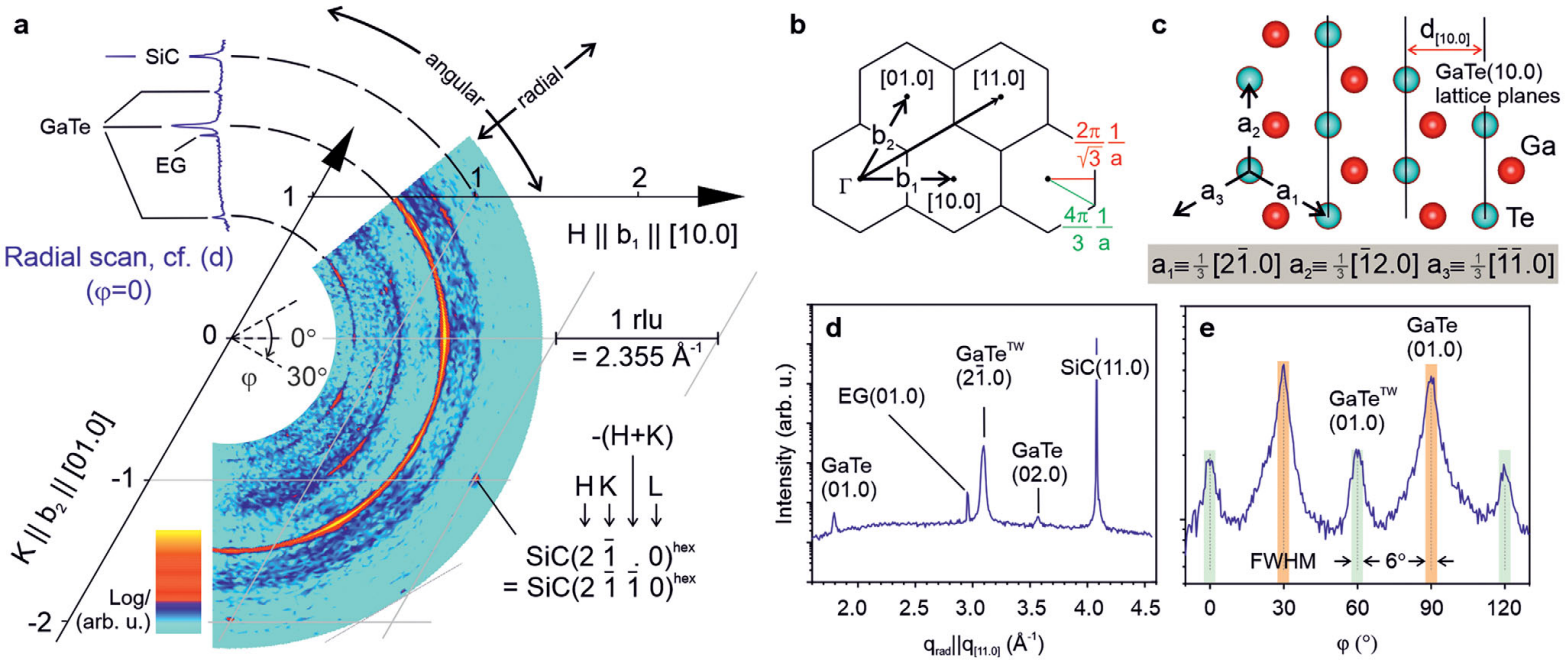
Impact of the EG/SiC substrate on the epitaxial registry for 2D h‐GaTe. a) In‐plane reciprocal space map depicting the azimuthal dependence of the (various) existing net plane distances. The scale is given in reciprocal lattice units (rlu) of SiC, where 1 rlu corresponds to 2.355 Å^−1^. b,c) are schemes of reciprocal and real space for GaTe, respectively. d) Radial scan intersecting the SiC(11.0) that proves the azimuthal alignment of the GaTe layer with respect to the EG substrate in the vdWH (see main text for further details). e) Azimuthal distribution along the most inner arc intersecting the GaTe(01.0) reflections with an indication for two sub‐domains rotated by 30° and a full width at half maximum (FWHM) of about 6°. Sample thickness: 27 ML.

To further understand the epitaxial registry of the h‐GaTe layers to the underlying EG/SiC substrate and to probe the crystallographic in‐plane lattice properties, we performed synchrotron‐based grazing incidence diffraction (GID) on a 27 MLs thick sample, capped with 30 nm of amorphous Si_3_N_4_ after the growth (see Experimental Section), at an incidence angle β_*i*_ = 0.20° close to the critical angle β_*c*_ for total external reflection (**Figure** **2**). In this situation, the amplitude of the incident X‐ray is exponentially damped into the crystal, turning this technique into a highly surface‐sensitive probe. However, compared to RHEED, which is near surface‐sensitive as it mainly probes the first few layers GaTe, GID allows the gathering of precise information about both the film and the underlying substrate. Due to the hexagonal symmetry of the substrate and GaTe layer, we chose a perfectly suitable coordinate system: H and K are the two in‐plane axes enclosing an angle of 60°, while L points perpendicular along the surface normal. Figure 2a depicts a large overview reciprocal space map within the horizontal (H,K)‐plane. Besides the intense SiC substrate reflections, emerging as well localized points, there are additional contributions from EG and GaTe. Generally, the intensity distribution along a particular arc represents the angular distribution of net planes with a lattice parameter defined by the diameter of the arc. In contrast, following the radial direction at a fixed azimuth probes different net plane distances oriented along this direction. Figure 2d,e are radial and azimuthal scans intersecting the SiC(11.0) and GaTe(01.0) reflections, respectively. The radial scan in Figure 2d shows two distinct GaTe orientations, the most dominant ${\mathrm{GaTe}}^{\mathrm{TW}}[2\bar{1}$.0] and a weaker GaTe[01.0] parallel to SiC[11.0], in agreement with the RHEED data. The lattice parameter obtained from the interplanar lattice distances for the ${\mathrm{GaTe}}^{\mathrm{TW}}[2\bar{1}$.0] and GaTe(01.0) orientations yields 4.05 ± 0.01 Å. This value matches the in situ RHEED data as a function of the deposition time of Figure 1b and highlights the effectiveness of the Si_3_N_4_ passivation layer for maintaining the pristine crystal information (the GID was taken a few weeks after air exposure). In addition, the EG layer is azimuthally 30° off the SiC substrate crystal since the EG(01.0) peak and the SiC(11.0) peaks appear in the same scan.^[^ ^28^, ^30^ ^]^ The angular inspection of the GaTe(01.0) confirms the results obtained by the in situ RHEED with a repetition every 60° and an epitaxial registry of the GaTe from the EG, given the symmetry relationship between the substrate and the growing layer.^[^ ^31^, ^32^ ^]^ Interestingly, the component 30° off is more present (20.8%) than in the case of GaTe on passivated silicon.^[^ ^24^ ^]^ This fraction is consistent with the azimuthal RHEED (Figure S3, Supporting Information), where approximately 22.6 ± 0.9%, 18.1 ± 0.2%, and 18.5 ± 0.2% are found for 9.7, 35, and 80 min deposition times, respectively. The stable ratio suggests that the epitaxial registry for GaTe is set in the first layers and remains unchanged throughout the growth process. The alignment of these domains could be the result of the remote epitaxy^[^ ^33^ ^]^ between the GaTe layer and 4H‐SiC driven by the stronger penetration of the potential field from the polar substrate in regions where the single‐layer EG is unable to fully screen the interaction. Although not observable using atomic force microscopy (AFM), we cannot completely exclude the presence of pinholes in graphene as the source of the enhanced interaction with the SiC substrate and the additional epitaxial registry^[^ ^34^, ^35^ ^]^ or defect‐mediated epitaxy.^[^ ^36^ ^]^ Further studies of GaTe as a function of EG thickness (e.g., using EG grown on vicinal SiC) and substrates with different ionicity^[^ ^37^, ^38^ ^]^ are needed to shed light on the causes of the greater presence of GaTe aligned to SiC and the role of the EG/SiC step region for the vdW epitaxy.

To confirm the crystal phase, micro‐Raman spectroscopy was performed (Figure S4, Supporting Information), revealing three narrow vibrational modes at 100.5 ($A_{1g}^{1}$), 172.1 ($E_{2g}^{1}$), and 287.1 cm^−1^ ($A_{1g}^{2}$), which are characteristic of the h‐GaTe.^[^ ^24^ ^]^ The decrease of the full width at half maximum (FWHM) of the $E_{2g}^{1}$ mode from 4.26 cm^−1^ for the deposition on silicon^[^ ^24^ ^]^ to 3.15 cm^−1^ is the evidence of the improved structural quality of the GaTe layer. In addition, the whole Raman spectrum shows no oxidation features, indicating the success of the passivation strategy for the GaTe/EG vdWH.

**Figure 3:**
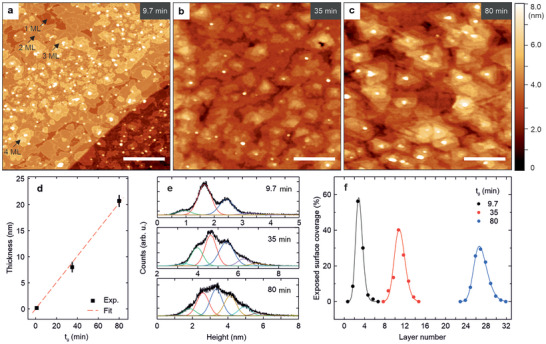
Growth dynamics from few to multiple layers 2D h‐GaTe. a–c) Atomic force microscopy (AFM) topography maps of the GaTe samples grown at *T*_*sub*_ = 445 °C for 9.7, 35, and 80 min (average thicknesses of 3, 11, and 27 MLs), respectively. Scale: 250 nm. The sample (a) was measured without the capping layer immediately after taking it out of the MBE chamber. d) Thickness of the GaTe layer as a function of *t*_*g*_. The red dashed line is a linear fit of the data to extract the growth rate. e) Height distributions obtained from an 8×8 µm^2^ area of (a–c), in which the single GaTe layers are visible and fitted with Gaussians. f) Exposed surface coverage, extracted from the area of the fit in panel (e), as a function of the number of layers of the GaTe surface for different deposition times. The solid lines are skewed Gaussians.

**Figure** **3** represents the evolution of the surface morphology as a function of deposition time. This analysis is crucial for evaluating the quality of the GaTe/EG interface and examining the nucleation and growth of both GaTe on EG and the subsequent GaTe on GaTe homoepitaxy. In particular, Figures 3a–c show the AFM from the epitaxial layer grown for 9.7, 35, and 80 min, respectively, corresponding to average thicknesses of 3, 11, and 27 MLs, as calculated from X‐ray reflectivity (XRR), X‐ray diffraction (XRD) and RHEED (Figure 3d; Figure S5, Supporting Information). As shown in Figure 3a, the first h‐GaTe layer grows fully coalesced on the EG substrate (see the dark brown regions) and corresponds to an assembly of Te‐Ga‐Ga‐Te^[^ ^8^ ^]^ and 0.8 nm in thickness.^[^ ^24^ ^]^ The second layer forms perfectly 2D grains although some small gaps are present. However, the lateral size of these islands is significantly larger than what is obtained on silicon for the same thickness.^[^ ^24^ ^]^ This is fully consistent with the higher crystalline quality indicated by the reduced FWHM of the Raman peaks (Figure S4, Supporting Information). Importantly, the islands extend laterally over the step, displaying the vertical decoupling typical of a vdW epitaxy due to the weaker interaction with the underlying EG/SiC substrate.^[^ ^39^, ^40^ ^]^ The sample grown for 9.7 min was left uncapped to avoid additional contribution to the surface roughness from the Si_3_N_4_ capping layer. This led to the oxidation of the edges and grain boundaries of the GaTe layers. As the material reacts to air its volume increases, causing the oxidized regions to rise in height (see the contour lines in the AFM map in Figure 3a). Based on the area enclosed by the oxidized boundaries, we estimate that the grown single‐crystalline islands are about 100 nm in diameter. Interestingly, starting from the third layer, the growth regime changes with an increased nucleation rate compared to the later growth rate. This leads to the formation of smaller islands on the top layer before the underlying ones are completed (see the arrows in Figure 3a and the height distributions in Figure 3e). By fitting each peak in Figure 3e using Gaussians, we determine the average GaTe layer thickness of 0.75 ± 0.06 nm and quantify the percentage of the surface that is covered by the exact layer height. Furthermore, we observe that the material tends to accumulate less toward step edges and we attribute it to the reduced chemical reactivity on the terraces of multilayer graphene with respect to ML graphene.^[^ ^28^ ^]^ As the deposition proceeds (case of 11 MLs in Figure 3b), the AFM data shows large coverage of the epitaxial layer with grains of 100–200 nm in diameter, but the 3D growth becomes more favorable with the tendency of the material to attach on the existing islands and to form stepped mounds. Finally, the thick sample of Figures 3c,e highlights the multilayer type of growth with the formation of multisteps and broadening of the height distribution, resulting in intensity modulation of the RHEED streaks normal to the surface (Figure S6, Supporting Information). This finding proves that layer‐by‐layer growth is achieved only for the first layer. As homoepitaxial growth progresses, the barrier to lateral diffusion and interlayer mass transport is increased due to the low surface energy of the atoms in this regime.^[^ ^41^ ^]^ We anticipate that larger atom diffusion^[^ ^39^ ^]^ via annealing steps mid‐growth,^[^ ^42^, ^43^ ^]^ and use of vicinal surfaces^[^ ^44^ ^]^ could improve the grain alignment and the coalescence process in order to maintain a continuous layer in the thicker regime. The small round features observed in the AFM maps do not increase in size throughout the growth and are tentatively attributed to parasitic 3D growth either at point defects or due to the coalescing of Ga into small droplets, but dedicated experiments are required to fully understand their nature. By plotting the surface area coverage as a function of layer number (the layer number is calculated by considering a growth rate of 0.25 nmmin^−1^, as extracted by the fit in Figure 3d), we find an increase of the roughness with the FWHM changing from 0.38 to 0.89 to 1.47 nm for the respective three thicknesses (Figure 3f). The coexistence of layers on the surface is typical for systems in which the interlayer mass transport is inhibited such that adatoms tend to nucleate on a layer at the impinging position, increasing the overall height (multilayer growth) instead of diffusing to layers below and creating a coherent layer by filling the channels.^[^ ^41^ ^]^ Further support for this interpretation is provided by the weakening of the RHEED intensity and the absence of GaTe oscillations as a function of deposition time (Figure S2, Supporting Information).

**Figure 4:**
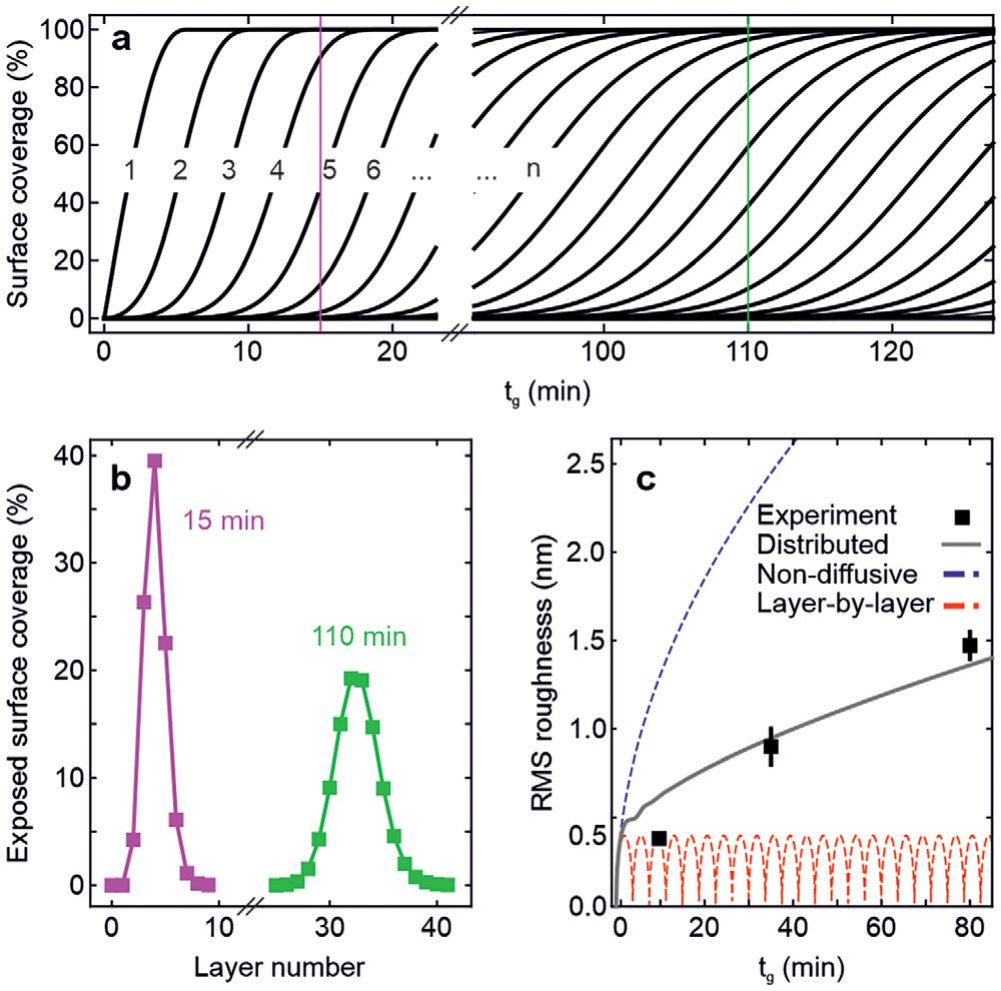
Interlayer mass transport from kinematic models. a) Surface coverage as a function of *t*_*g*_ for each layer (n), as simulated using Equation (1) (main text). b) Simulated exposed surface coverage as a function of layer number for *t*_*g*_ = 15 min (pink) and 110 min (green), as highlighted in (a). c) Root mean square (RMS) roughness of the grown material. The experimental data are extracted from the skewed Gaussian fits in Figure 3f. The dashed blue and red lines are the theoretical expected trends for purely non‐diffusive and layer‐by‐layer growths, respectively. The gray solid line is the RMS value as extracted from the simulation in (a) with the rate for adatoms transfer between layers *A*_*n*_ = 0.75 (see main text).

To extract further information about the growth dynamics and the adatom diffusion between layers, we compared the time evolution of the root mean square (RMS) roughness expected from kinematic models with the ones from the FWHM obtained by the measured surface coverage (see AFM data in Figure 3f). We employed two simplified growth models describing the extreme cases: the non‐diffusive one, where adatoms are confined to the layer they impinge on, resulting in 3D growth and high roughness, and the ideal layer‐by‐layer case, where adatoms can diffuse to the topmost unfilled layer filling it and leading to low roughness and smooth surfaces.^[^ ^45^ ^]^ Further details are provided in the Note S1 (Supporting Information). In addition, we applied a more refined distributed model in order to describe the intermediate roughness behavior. Our approach captures the island formation and growth and is implemented using the open‐source growth22 C++ code^[^ ^46^ ^]^ that serves as a “toy model” for the layer‐to‐layer transport process. This model assumes that adatoms landing on the *n*‐th layer have a probability α of transferring to the layer below. As a result, the growth is governed by a set of coupled differential equations describing the time evolution of the surface coverage θ_*n*_ of each layer with a growth rate of 1/τ:

(1) $$\frac{d\theta_{n}}{dt}=\frac{\theta_{n-1}-\theta_{n}}{\tau }+\frac{\alpha_{n}}{\tau }\left(\theta_{n}-\theta_{n+1}\right)-\frac{\alpha_{n-1}}{\tau }\left(\theta_{n-1}-\theta_{n}\right)$$

where

(2) $$\alpha_{n}=A_{n}\frac{d_{n}\left(\theta_{n}\right)}{d_{n}\left(\theta_{n}\right)+d_{n+1}\left(\theta_{n+1}\right)}$$

here, *A*_*n*_ represents the rate of adatom transfer between layers with values approaching 1 (0) in case of layer‐by‐layer (non‐diffusive) growths. By varying *A*_*n*_ we can thus continuously modulate between the two simpler models presented above. *d*_*n*_(θ_*n*_) is the perimeter of the *n*‐th layer, which can be defined as *d*_*n*_(θ_*n*_) = θ_*n*_(1 − θ_*n*_)^1/2^ by assuming that nucleation sites and island size increase with film thickness. The simulation of the surface coverage as a function of growth time is reported in **Figure** **4**a.

To ensure consistency with the experimental growth conditions, we used the growth rate parameters derived from XRR, XRD, and RHEED measurements (Figure 3d). For the sake of simplicity, we consider that neither the growth rate nor *A*_*n*_ change during deposition time and that the nucleation kinetics during heteroepitaxy and the subsequent homoepitaxy is the same. Whether this holds for the growth of GaTe on EG will be explored later in the text. Regardless, the nucleation regime constitutes only a small portion of the overall deposition process, thus having a limited impact on the rest of the growth and the simulation. In agreement with the experimental trends, the exposed surface coverage for two deposition times traced in Figure 4a exhibits an increase of the roughness with longer growths (Figure 4b). As shown in Figure 4c, the RMS roughness values extracted from the experimental data lie between both extremes of layer‐by‐layer and non‐diffusive growths. The *A*_*n*_ parameter of the distributed model was varied until the model matched the experimental data, returning *A*_*n*_ = 0.75 ± 0.1. This signifies partial interlayer transport and intermediate roughness.

**Figure 5:**
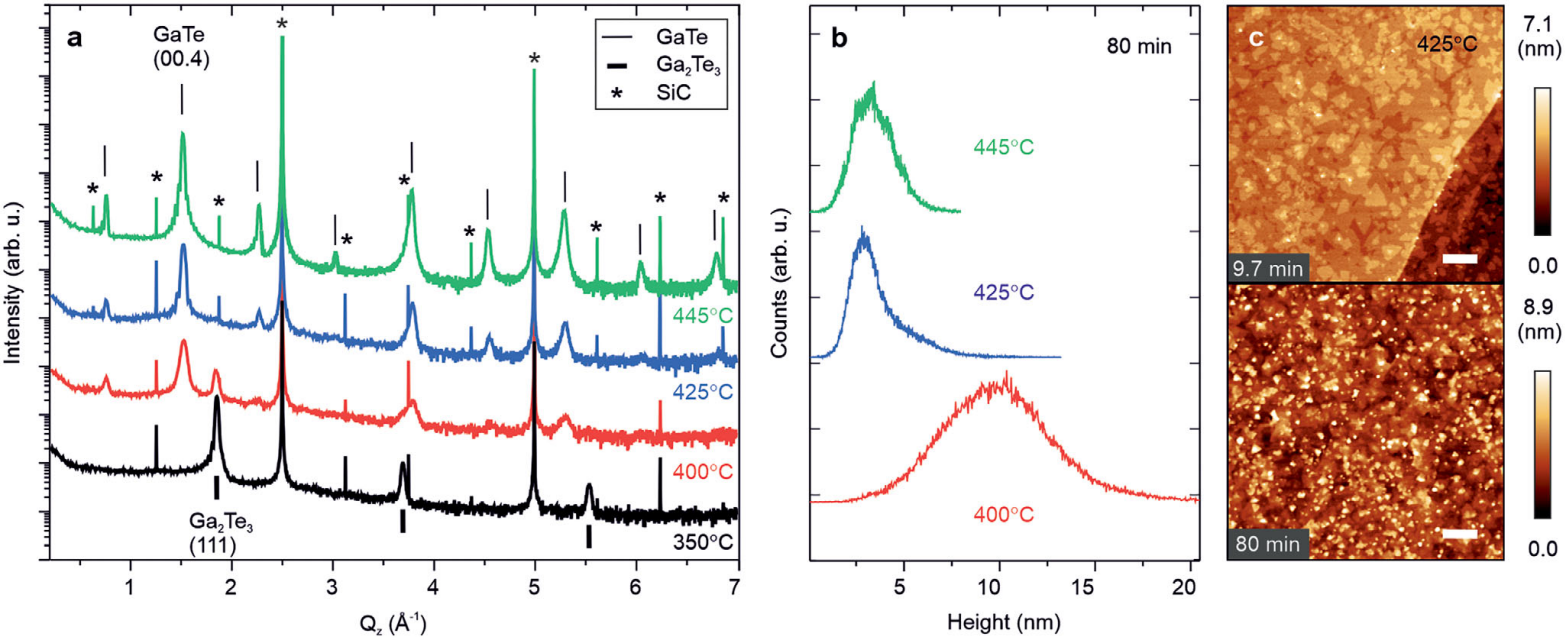
Phase diagram of GaTe as a function of substrate temperature. a) Symmetric out‐of‐plane ω‐2θ scan and b) height distribution of GaTe samples grown at *T*_*sub*_ = 350, 400, 425, and 445 °C and *t*_*g*_ = 80 min. c) AFM topography maps of the GaTe samples grown at *T*_*sub*_ = 425 °C for 9.7 min (top) and 80 min (bottom). Scale bar: 250 nm.

To gain more insight into the growth dynamics, we performed a series of depositions at different temperatures, namely *T*_*sub*_ = 350, 400, 425, and 445 °C. The symmetric XRD ω‐2θ scans along the [00.1] direction of the series at *t*_*g*_ = 80 min are presented in **Figure** **5**a.

At high growth temperature (445 °C) the peaks reveal that the layer is perfectly (00.1) oriented with the most pronounced order at reciprocal vector **Q**_*z*_ = 1.51 Å^−1^ from the (00.4) reflection of GaTe. The sharper Bragg reflections at **Q**_*z*_ = 2.50 ± 0.62 Å^−1^ are generated by the SiC substrate. The weaker SiC reflections, i.e., (00.5), (00.6), (00.7), arise from X‐ray double reflections.^[^ ^47^ ^]^ Interestingly, decreasing the temperature to 425 °C results in the emergence of a peak at **Q**_*z*_ = 1.85 Å^−1^, which is ascribed to the (111) reflection of Ga_2_Te_3_. Further lowering the growth temperature leads to increased Ga_2_Te_3_ content, until at 350 °C the only phase formed is Ga_2_Te_3_. This is attributed to a lower Te desorption at lower *T*_*sub*_.^[^ ^48^ ^]^ From the integrated intensity ratio of the GaTe(00.4) and Ga_2_Te_3_(111) we quantify the stoichiometry ratios of Ga and Te toward the 2:3 phase resulting in GaTe (Ga_2_Te_3_) of 88 (12)% and 99.5 (0.5)% at 400 °C and 425 °C, respectively.

The compositional shift is mirrored in the morphology of the grown surface (Figure 5b, see also the Raman data in Figure S7, Supporting Information). As the growth temperature is lowered from 445  °C, the surface loses its 2D layer‐by‐layer texture (see the previous discussion of Figure 3a), and the surface slope distribution derived from the topography maps broadens, as shown in Figure S8 (Supporting Information). A surface with fewer step‐edges is flatter and more homogeneous in terms of slopes compared to a more disordered surface, which is characterized by 3D features of varying island sizes. Specifically, the slope distribution of the 445 °C sample exhibits a multimodal profile, with distinct peaks at gradients of 0.014 and 0.043. These values corresponding to normal angles 0.83° and 2.47° are consistent with the flat and step‐edge regions of the GaTe surface, respectively. The absence of well‐defined features in the slope distribution for the samples grown at lower *T*_*sub*_ (400 and 425 °C) indicates higher surface disorder and less pronounced step‐edges. Despite the surface morphology shows increased disorder at lower temperatures, the height distribution remains similar to that at higher *T*_*sub*_ (425 and 445 °C, respectively). It is important to note that, while the disorder and surface roughness increase for longer deposition times with decreasing *T*_*sub*_, resulting in the formation of smaller islands, the temperature variations have minimal impact on the nucleation regime and GaTe layer homogeneity on the underlying EG, as shown by comparing the surface morphologies at 425 °C after 9.7 (top) and 80 (bottom) min growth (Figure 5c) with the equivalent AFM images at 445 °C (Figures 3a,c). The findings indicate that the EG surface facilitates uniform and planar nucleation across the temperature range, in contrast to the roughening during the later stages of GaTe homoepitaxy. This observation suggests that different parameters govern heteroepitaxy since surface energy, lattice constant, and polarity are expected to change for the two distinct interfaces (GaTe/EG, GaTe/GaTe). The shift toward disordered 3D growth at lower temperatures is consistent with reduced adatom mobility as well as interlayer mass transport. In addition, the increase in parasitic Ga_2_Te_3_ content is likely to contribute to a more disordered morphology (Figure S9, Supporting Information).

**Figure 6:**
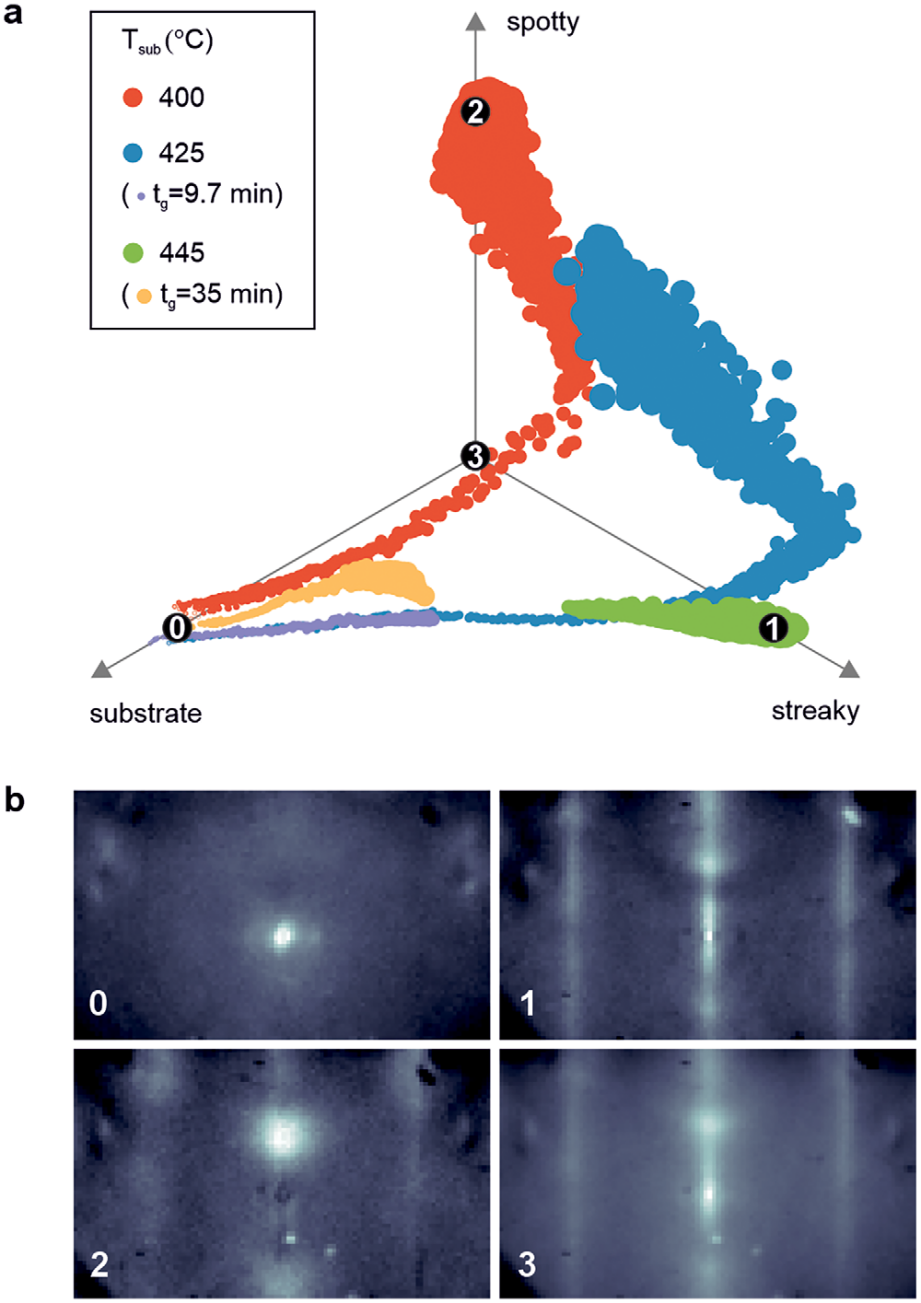
Contrastive principal component analysis of RHEED data. a) Projection of the 5D cPCA‐analyzed time‐ and temperature‐dependent RHEED data onto three physically meaningful vectors defining the dominant patterns in the dataset (see main text). The size of the data points represents the time variable. When not specified, all the samples have *t*_*g*_ = 80 min (green sample at *T*_*sub*_ = 445 °C is shown only after 14 min from the start of the growth). The numbers 0–3 indicate the positions from which the images in (b) were sampled. b) Mean representative images for the clusters 0–3 in (a) acquired perpendicular to the [10.0] direction.

In situ RHEED can be used to track changes in surface quality during the heteroepitaxy and subsequent homoepitaxy. However, the manual interpretation of RHEED patterns for identifying in real‐time during growth the 2D‐3D transition, which manifests in changes in surface structure, is challenging and might introduce subjective biases. To overcome these issues, we performed cPCA from five separate GaTe deposition runs: 400 °C (80 min), 425 °C (9.7, 80 min), and 445 °C (35, 80 min), as shown in **Figure** **6**a.

PCA has previously been applied to MBE growth to identify distinct trends in the evolution of RHEED patterns and facilitate the classification of different growth modes.^[^ ^49^, ^50^, ^51^ ^]^ When working with RHEED, one challenge is the reproducibility of diffraction patterns, which depends highly on the sample's position in space. Variations in the electron incidence angle, shifts in the pattern position on the phosphor screen, obstructions in the optical path, and fluctuations in beam focus and intensity can introduce high‐variance directions when performing PCA, potentially leading to inaccurate results. In particular, the starting conditions, such as the RHEED pattern of the substrate, should remain as consistent as possible, with minimal spread in PCA space. To address this issue, it is important to filter out the variance arising from these extrinsic factors and focus only on pattern‐specific features. To this end, we employ cPCA, a method that constructs a PCA subspace of a target dataset by minimizing the variance of a user‐defined background that captures the unwanted variations while maximizing the variance of the actual target dataset (Notes S2–3, Supporting Information).^[^ ^52^ ^]^ Specifically, cPCA computes the eigenvectors of the cumulative variance:

(3) $$C=C_{x}-\alpha C_{y}$$

where *C*_*x*_ and *C*_*y*_ are the covariance matrices of the target and background datasets, respectively, and α is a parameter that controls the trade‐off between preserving the target variance and reducing background variance. At the extremes, α = 0 results in standard PCA, while increasing α progressively suppresses the background variance. In the limit of α = ∞, only the directions orthogonal to the component that minimizes the background variance remain.

In our study, cPCA was applied by treating individual RHEED patterns as a single data point, and the first five principal components were retained. We use RHEED images of the initial SiC/EG substrate as the background dataset, ensuring that PCA components focus on growth‐related variations. α was chosen to maximize the retained variance while minimizing the spread of the substrate RHEED patterns in the cPCA‐space. Specifically, we introduced the spread *d*_*RMS*_ as the RMS distance from the centroid of the substrate data (*d*_0_ for the original dataset), the cumulative explained variance ratio for *N* components Λ_*N*_ and defined a fitness function, Λ_*N*_/(*d*_*RMS*_/*d*_0_), which, when maximized, results in the desired outcome (Note S3, Supporting Information). The first five components together account for 54% of the total variance in the dataset (Figures S10– S11, Supporting Information). To visualize the latent space in physically meaningful terms, we selected three representative mean 5D vectors from our PCA dataset, corresponding to the three primary pattern types observed: i) SiC/EG substrate (growth start), ii) spotty GaTe (end of the 400 °C growth), and iii) streaky GaTe (end of the 445 °C and 80 min growth). Figure S12 (Supporting Information) shows the projection of the 5D space onto the new subspace. Visual inspection reveals that the dataset is largely constrained to the plane defined by the unit vectors, allowing us to project the data to 2D, as shown in Figure 6a. The vertices of the plot correspond to the unit vectors of the physical basis, the marker size is proportional to deposition time, and the color denotes the specific growth run. Figures S13‐S14 (Supporting Information) show that the original data and its reconstruction obtained by inverting the dimensionality reduction exhibit excellent agreement. This indicates that the retained components and the subsequent projections effectively capture the latent structure of the data with minimal information loss. The labels in Figure 6a identify four distinct regions within the processed dataset. All growths begin in the substrate corner of the graph (region 0). A representative pattern reconstructed by sampling from this region is shown in Figure 6b. During the initial period of growth (2–3 min), all samples exhibit similar trajectories in the lower‐dimensional latent space. However, RHEED patterns from samples grown at 445 °C move to the corner that describes streaky patterns (region 1), whereas those grown at 425 and 400 °C converge toward the spotty corner (region 2). As shown in Figure 6b, the image reconstructed from region 1 reveals streaky patterns (indicative of flat surfaces). The convergence of samples grown at the highest temperature to this region suggests that, at this temperature, there is a tendency for the surface to remain flat throughout the entire growth process. However, the samples grown at 400 and 425 °C initially exhibit streaks (see patterns reconstructed from regions 1 and 3 in Figure 6b), but then drift to region 2 with a spotty pattern, indicative of 3D growth. Notice that the beginning of the growth at 425 °C follows exactly the trajectory traced at 445 °C, but then turns toward the spotty region. This behavior is consistent with the planar growth observed after a short growth run in the AFM data of Figure 5c. Overall, the trajectories observed in the cPCA analysis of the RHEED data perfectly align with the roughening observed in the AFM data. These trends can be made clearer by plotting the contribution from each one of the three physically interpretable vectors, as shown in Figure S15 (Supporting Information). The samples grown at low *T*_*sub*_ (400 and 425 °C) have a peak in the streaky component, but this is suppressed as the data becomes more and more spotty. For higher *T*_*sub*_ (445 °C), the data steadily converges toward high levels of streakiness.

In summary, by reducing the dimensionality of the RHEED dataset using cPCA, we extract trends that reproduce the results observed in the AFM data, namely that higher temperatures and shorter deposition times correspond to a greater tendency toward flat, 2D growth. The initial heteroepitaxy of GaTe on EG promotes 2D growth across a wide temperature range, suggesting that the interaction with the substrate controls nucleation rather than the temperature‐dependent surface mobility of the adatoms. However, the subsequent homoepitaxy follows a different growth regime. Further studies are necessary to better understand the mechanisms behind the heteroepitaxy as well as the transition from a 2D to a more 3D growth after the deposition of the initial GaTe layer.

## 3 Conclusion

In conclusion, we demonstrated the all‐epitaxial h‐GaTe/EG vdWH. Structural, morphological, and optical characterization provided evidence for the formation of large coverage and strain‐free 2D‐layered GaTe due to its weak vdW interaction with the underlying EG layer. The appearance of rotated in‐plane domains indicates the remote interaction between the GaTe layer and SiC, in contrast with the mostly aligned 2D GaTe along the highest symmetry direction of the EG. We observed complete coalescence of the first h‐GaTe layer, followed by a multilayer‐type growth in the subsequent homoepitaxy. A distributed growth model, which accounts for the interlayer diffusion of adatoms, effectively describes the epitaxial regime that lies between non‐diffusive and layer‐by‐layer growth. By varying the substrate temperature from 445 to 350 °C, the GaTe surface roughens due to reduced surface diffusion, and its stoichiometry changes from 1:1 to 2:3, as the desorption rate of Te reduces. These findings highlight the urge for advanced growth strategies for the synthesis of vdW materials that increase lateral growth and mass transport between layers. From the statistical pinpoint via the cPCA approach of RHEED data as a function of both deposition temperature and time, we track in situ the change in the growth kinetics and define the boundaries for the layered regime. This experimental‐computational approach can be extended to other epitaxial vdWHs to study novel 2D systems via the information on phase diagrams and growth dynamics extracted by analytical tools. We envision the few‐layers GaTe/EG vdWH showing advantages for thermoelectric and photodetection applications. Notably, the investigated system presents an exciting opportunity for device integration as the heteroepitaxy enables the creation of functional 2D devices consisting of semiconductors and conductors by reducing the number of additional fabrication steps.

## 4 Experimental Section

### Molecular Beam Epitaxy

GaTe films were grown by solid‐source MBE on EG, which was obtained by surface graphitization of 1 cm^2^ semi‐insulating 4H‐SiC(00.1) substrates with a miscut of less than 1%. The fabrication and characterization of the initial EG used in this work were described in detail elsewhere.^[^ ^53^, ^54^ ^]^ Before loading the substrates into the growth chamber, they were subjected to outgassing at 350 °C in a separate chamber. Subsequently, elemental Ga and Te were evaporated from Knudsen cells, with a Te/Ga flux ratio of approximately 40 to account for the low sticking coefficient of Te on the graphene surface.^[^ ^55^ ^]^ To enhance heat transfer efficiency, the substrates were coated with approximately 1 µm of Ti on the reverse side via electron beam evaporation. *T*_*sub*_ was maintained at 455 °C for all the layered samples throughout the growth. In situ growth monitoring was conducted using RHEED. Given the high reactivity of the GaTe surface to air, the films were capped by sputtering with approximately 30 nm of amorphous Si_3_N_4_ at room temperature in UHV in order to promote the conformal growth.

### Atomic Force Microscopy and Raman Spectroscopy

The surface morphology of the EG and GaTe films was investigated via tapping mode AFM (Bruker Multimode). The presence of the conformal capping layer for the thicker GaTe films slightly reduces the sharpness of the AFM contrast without impacting on the analysis. Micro‐Raman spectroscopy (spot size of ≈1 µm) was carried out in backscattering configuration by means of a continuous‐wave (CW) laser with excitation wavelengths at 473 and 632.8 nm. The symmetry of the vibrational modes was assigned through polarization‐resolved spectroscopy.

### X‐ray Diffraction

To investigate the lattice properties of GaTe along the growth direction and the azimuthal dependence of various in‐plane lattice parameters, synchrotron‐based coplanar XRD and GID was employed, respectively. Corresponding experiments were conducted at the SpLine beamline BM25B at the European Synchrotron Radiation Facility (ESRF) in Grenoble, utilizing an X‐ray energy of 18 keV equivalent to an X‐ray wavelength of λ = 0.6888 Å. The layer thicknesses were estimated by high‐resolution XRD and XRR using a four‐circle PANalytical X'Pert Pro Materials diffractometer, equipped with a Ge(220) hybrid monochromator providing Cu‐Kα_1_ radiation with a wavelength of λ = 1.5406 Å. In the case of ω‐2θ scans, a 1 mm slit was employed at the detector side.

### Contrastive Principal Component Analysis

cPCA was used to reduce the dimensionality of the high‐volume RHEED dataset collected during the GaTe growth process, thereby facilitating the extraction of key features from the time‐resolved diffraction patterns. cPCA analysis was performed using the Python implementation available from the authors of Ref. [52]. Each RHEED image was treated as an individual data point in the principal component (PC) space.

## Conflict of Interest

The authors declare no conflict of interest

## Supporting information

Supporting Information

## Data Availability

The data that support the findings of this study are available from the corresponding author upon reasonable request.
