# Supplementary material for: Van der Waals Epitaxy of 2D Gallium Telluride on Graphene: Growth Dynamics and Principal Component Analysis
Source: Small. 2025 May 2;21(24):2503993. doi: 10.1002/smll.202503993 (PMC12177854; doi:10.1002/smll.202503993)
Supplement: Supplementary file 1 — Supporting Information [file SMLL-21-2503993-s001.pdf]

NANO · MICRO
small

Supporting Information

for *Small*, DOI 10.1002/smll.202503993

Van der Waals Epitaxy of 2D Gallium Telluride on Graphene: Growth Dynamics and Principal Component Analysis

*Michele Bissolo, Michael Hanke, Raffaella Calarco, Jonathan J. Finley, Gregor Koblmüller, J. Marcelo J. Lopes and Eugenio Zallo**

Supporting Information

Van der Waals Epitaxy of 2D Gallium Telluride on Graphene: Growth Dynamics and Principal Component Analysis

*M. Bissolo,[1] M. Hanke,[2] R. Calarco,[3, 2] J. J. Finley,[1] G. Koblmüller,[1,4] J. Marcelo J. Lopes,[2] and E. Zallo[1, 2, *]*

[1)]Walter-Schottky-Institut and TUM School of Natural Sciences, Technische Universität München, Am Coulombwall 4, 85748 Garching, Germany

[2)]Paul-Drude-Institut für Festkörperelektronik, Leibniz-Institut im Forschungsverbund Berlin e.V., Hausvogteiplatz 5-7, 10117 Berlin, Germany

[3)]Institute for Microelectronics and Microsystems (IMM), Consiglio Nazionale delle Ricerche (CNR), Via del Fosso del Cavaliere 100, 00133 Rome, Italy

[4)]Institute of Solid State Physics, Technical University Berlin, Hardenbergstrasse 36, 10623 Berlin, Germany

**E-mail: eugenio.zallo@tum.de*

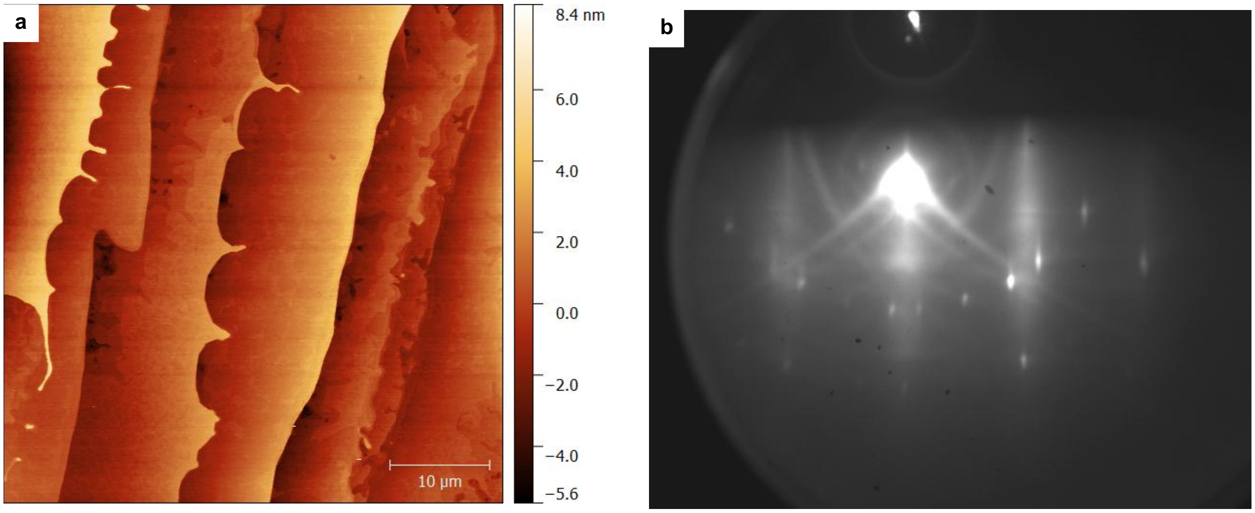

Figure S1: AFM and RHEED images of EG on SiC substrate. (a) AFM map of graphitized SiC substrate surface. Epitaxial graphene grows as monolayer (ML) on the terraces and few-layers at the step edges. [1] **(**b) RHEED pattern along the SiC(11.0) or EG(10.0) direction. The surface morphology shows the SiC terraces, originating from a small miscut angle (<1%), covered by epitaxial graphene layers. The RHEED pattern of EG on SiC, obtained by surface graphitization of semi-insulating 4H-SiC(0001) confirms high surface quality.

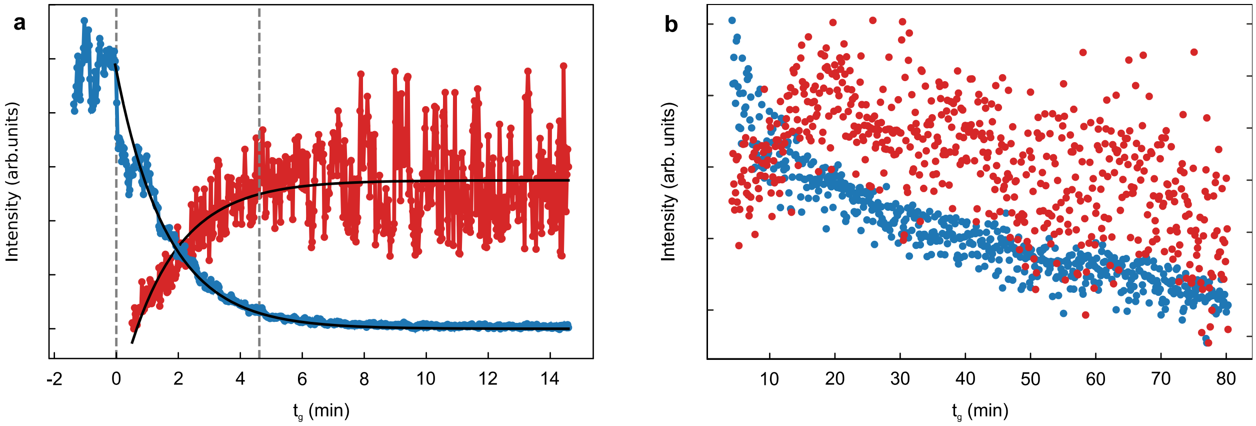

Figure S2: Time evolution of RHEED intensity. (a) RHEED intensity of SiC[10.0] (blue) and $GaTe^{TW}$[10.0] (red) as a function of growth time (t_g). The continuous black lines are exponential fits. The vertical dashed grey lines indicate the start of the growth and the completion of the first layer, according to the growth rate extracted from the fits. The intensity of the SiC[10.0] streak as a function of time exhibits an almost perfectly exponential dampening of the diffraction signal. Simultaneously, the $GaTe^{TW}$[10.0] streak appears, increasing in brightness at the same rate as the SiC streak dampens. Using the known chamber geometry (a 5° grazing incidence angle of the electron beam) and electron energy (from the accelerating voltage of 15 kV), and assuming an inelastic mean free path (IMFP) of 6.61 nm, we fit both curves to an exponential function and obtain a growth rate of 0.1756 nm/min. This value is consistent with XRD oscillations and XRR results. (b) RHEED intensity of the primary streak (blue) and $GaTe^{TW}$[10.0] for longer growth times.

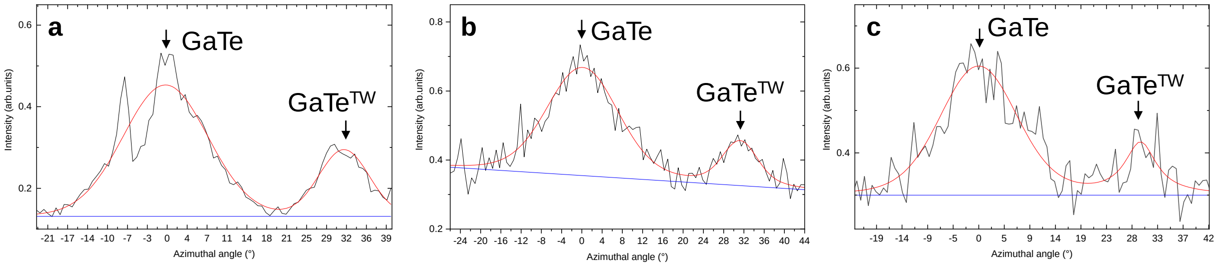

Figure S3: Azimuthal RHEED of thickness-dependent GaTe. (a-c) GaTe[01.0] diffraction peaks from an azimuthal RHEED scan for samples grown for 9.7 (a), 35 (b), and 80 (c) min. The GaTe[01.0] and $GaTe^{TW}$[10.0] peaks extracted from the azimuthal scans indicate the presence of 20% of $GaTe^{TW}$ on the surface. The value does not change as a function of deposition time (see main text).

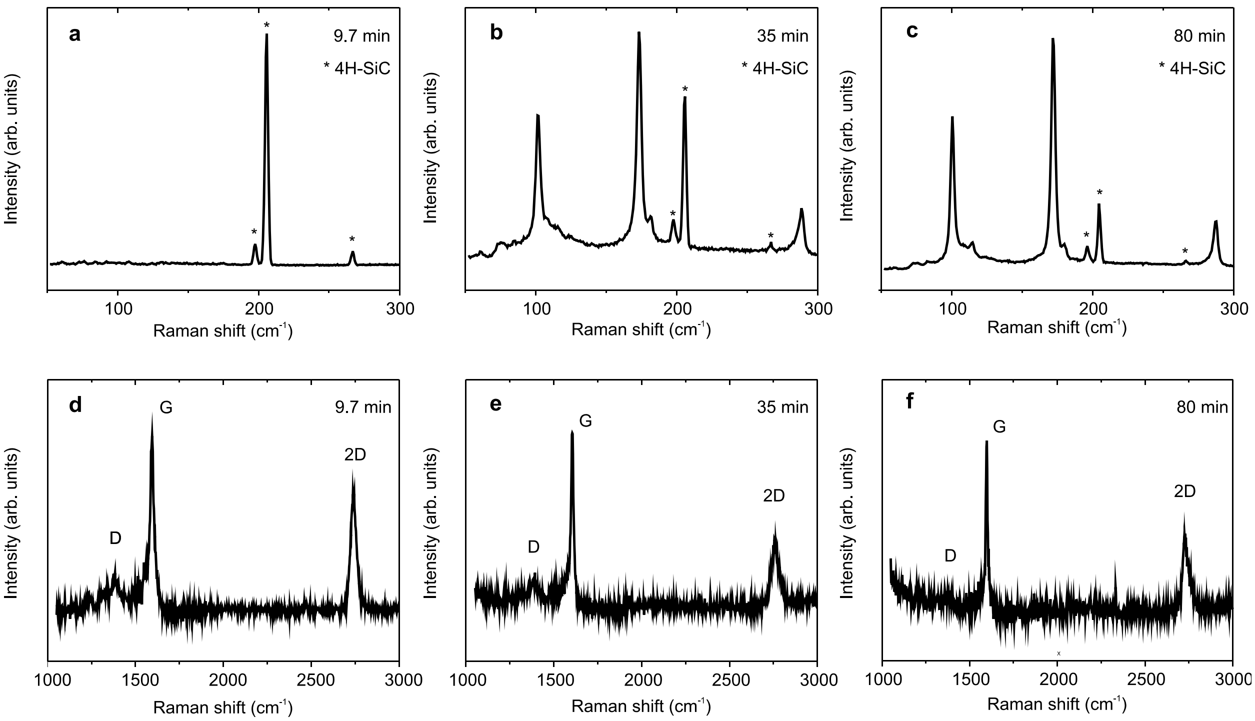

Figure S4: Raman of thickness-dependent GaTe. Raman spectra of h-GaTe after 9.7, 35, and 80 min deposition time (3, 11, and 27 MLs, respectively) in the low (a-c) and high (d-f) frequency regions with a 473 nm laser excitation. Due to the small film thickness, the Raman spectrum in (a) shows almost no signal. The samples in (b) and (c) show comparable spectra with well-defined peaks and narrow linewidths (~3 cm^{-1} for the 172.1 cm^{-1} mode). The higher wavenumber region of (d-f) highlights the high structural quality of the vdWH and the absence of rehybridization in the EG layer. This is testified by unaltered graphene-related G and 2D peaks and no increase in the defect-related D peak after GaTe deposition. The shift and change of width of the 2D peak in the 80 min sample can be the effect of the different graphene thicknesses at the step edges. [1] The spectra from (d-f) are obtained after subtraction of the second-order Raman of 4H-SiC.

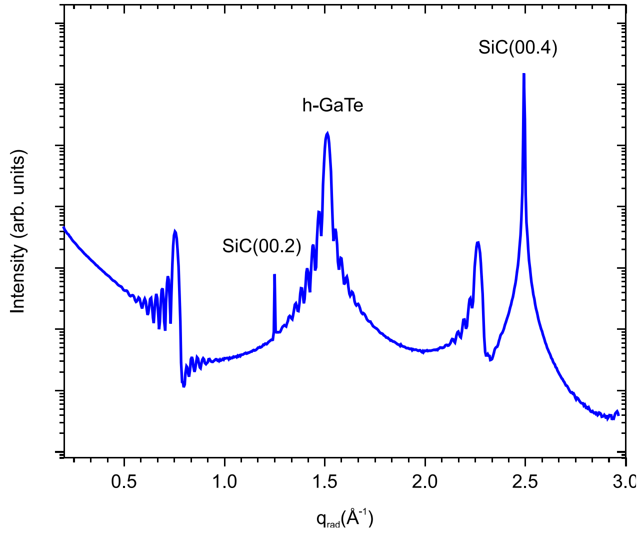

Figure S5: Synchrotron-based XRD. Symmetric out-of-plane 2θ-ω scan of 27 ML thick GaTe. The h-GaTe sample is characterized by narrow peaks and prominent oscillations (thickness of 21.7±0.1 nm), indicating high crystalline quality and smooth interfaces.

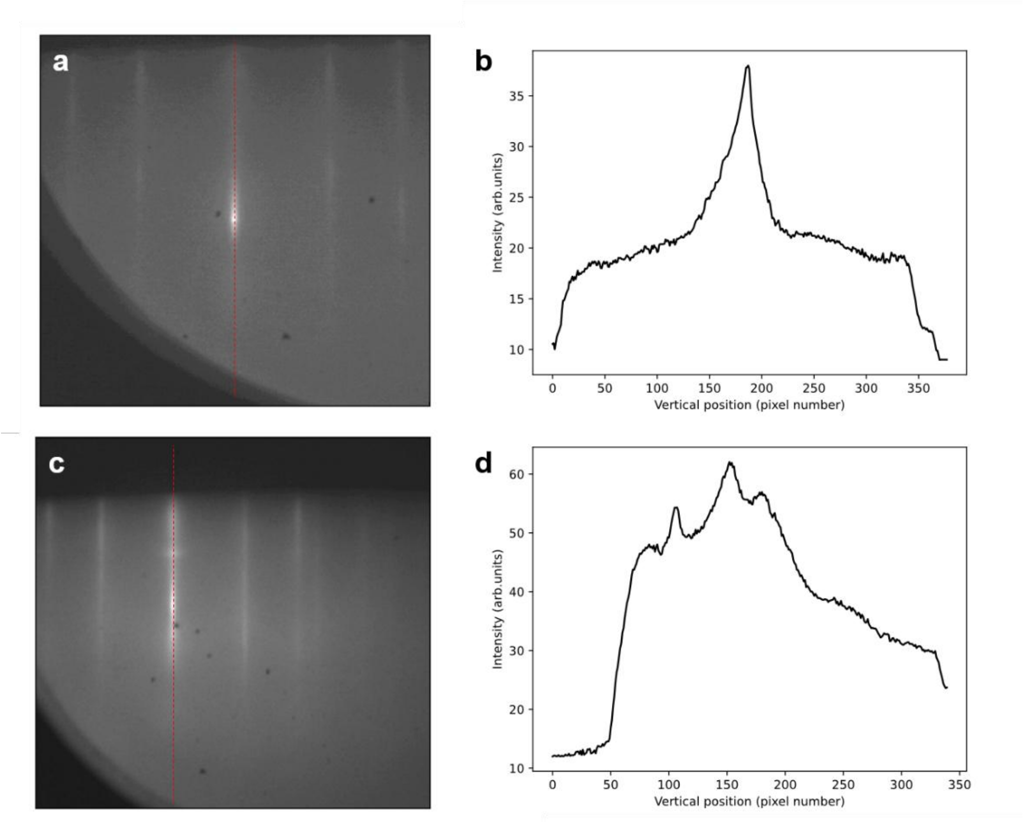

Figure S6: RHEED streak intensity modulation. (a) RHEED pattern of GaTe after 30 min of growth. (b) RHEED intensity along the vertical dashed red line in (a). (c) RHEED pattern of GaTe after 80 min of growth. (d) RHEED intensity along the vertical dashed red line in (d). The modulation of the RHEED intensity along the central primary streak, observed after longer deposition times, indicates the roughening of the surface.

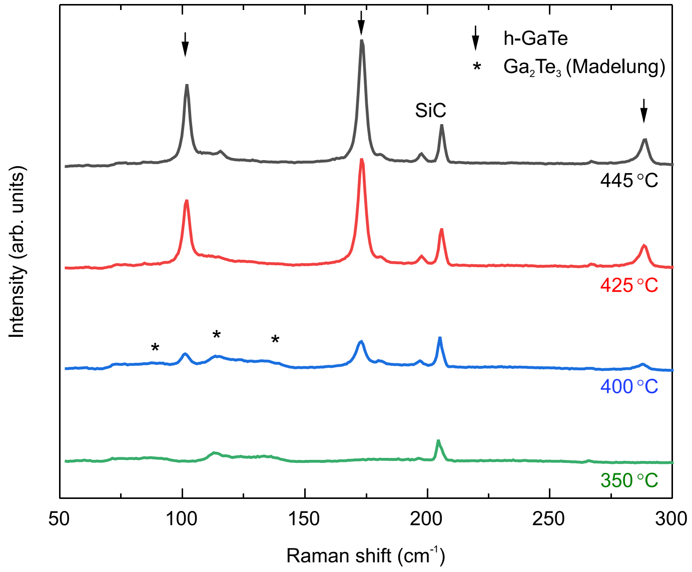

Figure S7: Growth temperature-dependent Raman. Raman spectra for different GaTe deposition temperatures (350, 400, 425, and 445 °C) in the low-frequency regions with a 473 nm laser excitation. The arrows indicate the peaks associated with h-GaTe, while the stars denote the peaks arising from the parasitic Ga_2Te_3 growth. The change in composition observed in the XRD data (see main text) is also reflected in the Raman spectrum. As the temperature is decreased, the Raman peaks of h-GaTe disappear, while new peaks characteristic of Ga_2Te_3 appear.

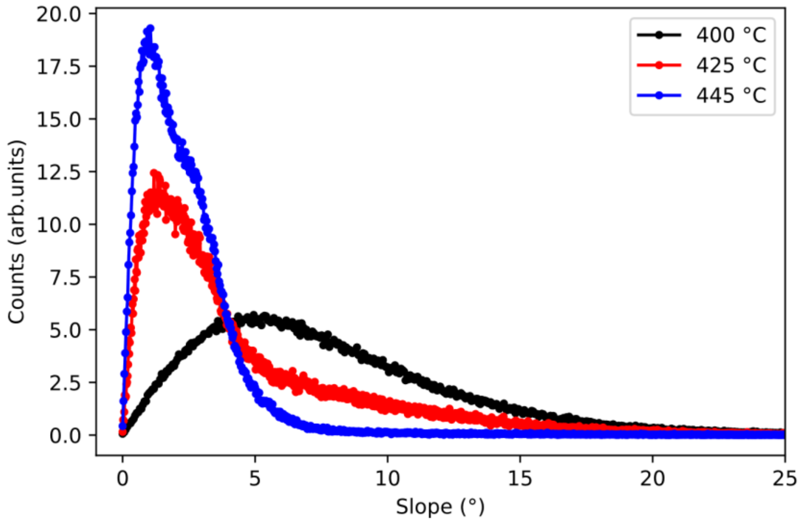

Figure S8: Surface slope distribution. Surface slope distribution as extracted from AFM maps taken at 400, 425, and 445 °C (black, red, and blue, respectively). The slope distribution of the samples grown at different temperatures broadens and shifts to larger slopes as the temperature diminishes. The blue curve, corresponding to a layered h-GaTe, is characterized by a peak with a shoulder. The peak corresponds to the flat regions of the surface, while the shoulder consists of the oxidized step edges of the GaTe layers.

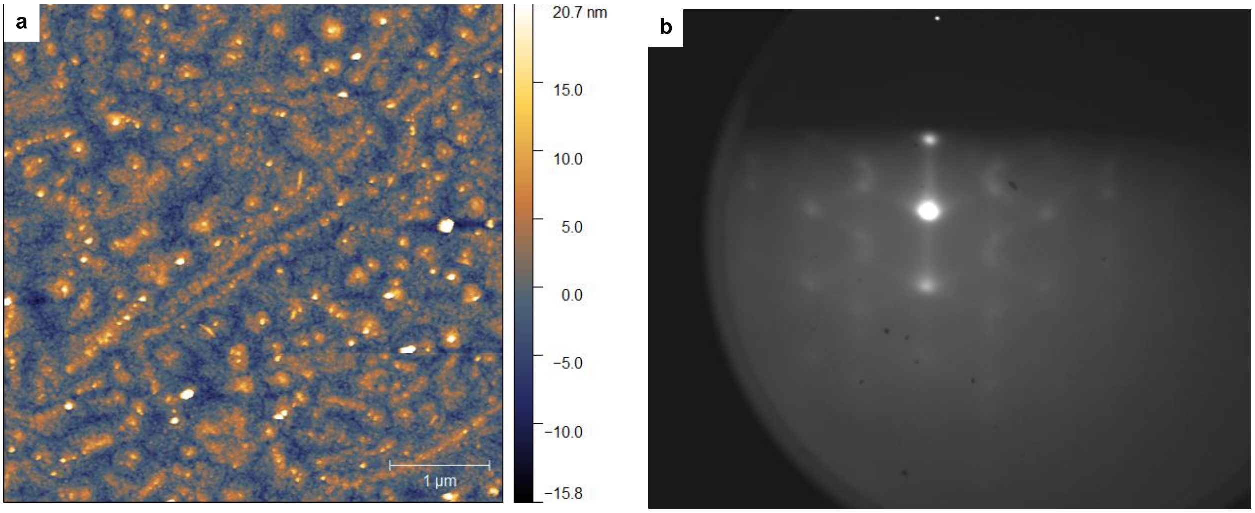

Figure S9: AFM and RHEED of Ga_2Te_3. (a) Topography map resulting from the mixed GaTe and Ga_2Te_3 growth at 400 °C, and (b) the corresponding RHEED pattern. The RHEED pattern is spotty, characteristic of a 3D surface morphology. The AFM map confirms the high surface roughness.

Note S1 : Models for non-diffusive and layer-by-layer growth [2]

The layer-by-layer model of growth mentioned in the main text assumes that atoms fill the topmost layer until it is complete. The surface coverage increases at a rate of $1/\tau$ ML per second, and can be written as $\theta_0 = 1$, $\theta_1 = t/\tau$, $\theta_n = 0$ for $n > 1$, such that only one actively growing layer exists on the surface at one time.

In contrast, the non-diffusive growth model describes growth conditions in which adatoms are confined to the layer they impinge on due to a diffusion barrier across step edges. This leads to rapid growth on large, exposed layers, and slower growth on nearly completed ones, and therefore multilayer, 3D growth. The growth rate for this model, also at $1/\tau$ MLs per second, is described by the following equation:

$d\theta_n/dt = (1/\tau)\,(\theta_{n-1} - \theta_n)$, with $\theta_0(t) = 1$ and $\theta_n(0) = 0$, which can be solved analytically with a surface coverage $\theta_n(t) = 1 - e^{(-t/\tau)} \sum_{j=0}^{n-1} (t/\tau)^j \,/\, j!$.

The root mean square (RMS) roughness (Δ^2) for both models can be computed using:

$\Delta^2 = \sum_{n=0}^{\infty} (n - t/\tau)^2\,(\theta_n - \theta_{n+1})$.

Note S2: Contrastive PCA

Principal Component Analysis (PCA) is widely used for dimensionality reduction and data exploration, but it does not always prioritize the most relevant variations in a dataset. Contrastive PCA (cPCA) provides an alternative in cases where background noise or dominant but uninformative variations obscure meaningful patterns. The unwanted variance is suppressed by obtaining a "background" dataset that captures the uninteresting variations (e.g., noise, artifacts, or experimental fluctuation), which is compared with the "target" dataset of interest that contains both the unwanted variance and the interesting variations. This is achieved by redefining the covariance matrix, from which PCA usually computes the eigenvectors. Given a "target" dataset with covariance matrix $\boldsymbol{C}_{\mathrm{x}}$ and a "background" dataset with covariance matrix $\boldsymbol{C}_{\mathrm{y}}$, cPCA computes the eigenvectors of the following modified covariance matrix: $\boldsymbol{C} = \boldsymbol{C}_{\mathrm{x}} - \boldsymbol{\alpha}\boldsymbol{C}_{\mathrm{y}}$, where α is a hyperparameter controlling the trade-off between preserving variance in the target dataset and reducing variance from the background dataset. For α=0, cPCA reduces to standard PCA, as the background dataset is ignored. As α increases, cPCA prioritizes more and more components that also minimize the background variance to the point at which the target variance is ignored; by choosing the right α, cPCA identifies directions in feature space where the target dataset has high variance, but the background dataset has low variance. This enhances the discovery of meaningful trends unique to the target dataset. For a more detailed explanation of cPCA, see Ref. [3].

In our case, the background used is the initial RHEED images of the various growth runs. To increase the number of samples, we augment the dataset by mirroring the RHEED patterns, and slightly translating their center by a few pixels.

Mathematical formulation of the projection from the 5D space onto the 2D space

Step 1: Projection from 5D to 3D

We define three representative **5D** vectors $\boldsymbol{v_1}, \boldsymbol{v_2}, \boldsymbol{v_3}$ from the dataset, which correspond to distinct physical states (substrate, streaky and spotty). We then separately project each datapoint $\boldsymbol{x} \in \mathbb{R}^{\mathbf{5}}$ onto the three $\boldsymbol{v_i} \in \mathbb{R}^{\mathbf{5}}$, obtaining a **3D** vector $\boldsymbol{x'} = (\boldsymbol{x} \cdot \widehat{\boldsymbol{v}}_{\mathbf{1}})\widehat{\boldsymbol{v}}_{\mathbf{1}} + (\boldsymbol{x} \cdot \widehat{\boldsymbol{v}}_{\mathbf{2}})\widehat{\boldsymbol{v}}_{\mathbf{2}} + (\boldsymbol{x} \cdot \widehat{\boldsymbol{v}}_{\mathbf{3}})\widehat{\boldsymbol{v}}_{\mathbf{3}}$. This process can be written as a projection

$$\boldsymbol{X'} = \boldsymbol{D}\boldsymbol{V}^{\boldsymbol{T}}\boldsymbol{X},$$

where $\boldsymbol{V} = [\boldsymbol{v_1}, \boldsymbol{v_2}, \boldsymbol{v_3}]$, thus the **5×3** matrix whose columns are $\boldsymbol{v_1}, \boldsymbol{v_2}, \boldsymbol{v_3}$**,** and $\boldsymbol{D}$ the 3×3 diagonal matrix containing the normalization factors $\frac{\mathbf{1}}{\boldsymbol{v_i}^{\boldsymbol{T}}\boldsymbol{v_i}}$.

Step 2: Normalization

The projected dataset $\boldsymbol{X'}$ is then normalized, such that $\boldsymbol{X'} \in [\mathbf{0}, \mathbf{1}]$ with

$$\boldsymbol{X'}_{\mathbf{norm}} = \frac{\boldsymbol{X'} - \mathbf{X'}_{\boldsymbol{min}}}{\mathbf{X'}_{\boldsymbol{max}} - \mathbf{X'}_{\boldsymbol{min}}},$$

where $\mathbf{X'}_{\boldsymbol{min}}$ and $\mathbf{X'}_{\boldsymbol{max}}$ are the column-wise minimum and maximum values.

Step 3: Projection from 3D to 2D

The dataset is projected onto the plane spanned by the vertices of the standard basis in **3D** $\mathbf{e}_{\boldsymbol{i}}$ with normal vector $\boldsymbol{n} = \frac{\mathbf{1}}{\sqrt{\mathbf{3}}}(\mathbf{1}, \mathbf{1}, \mathbf{1})$. The projection matrix can then be constructed as $\boldsymbol{P} = (\boldsymbol{I} - \boldsymbol{n}\boldsymbol{n}^{\boldsymbol{T}})$, such that

$$\boldsymbol{X''}_{\boldsymbol{3D}} = \boldsymbol{X'}_{\boldsymbol{norm}}\boldsymbol{P}.$$

We then define an orthonormal basis $\boldsymbol{u}_{\mathbf{1}} = \frac{e_1 - e_3}{\|e_1 - e_3\|}$ and $\boldsymbol{u}_{\mathbf{1}} = \frac{(e_2 - e_3) - \langle e_2 - e_3, \boldsymbol{u}_1 \rangle \boldsymbol{u}_1}{\|(e_2 - e_3) - \langle e_2 - e_3, \boldsymbol{u}_1 \rangle \boldsymbol{u}_1\|}$ spanning the **2D** plane. The dataset projected onto the plane is then expressed in **2D** via the new basis using

$$\boldsymbol{X''}_{\mathbf{2D}} = \boldsymbol{X''}_{\mathbf{3D}}\boldsymbol{U},$$

where $\boldsymbol{U} = [\boldsymbol{u}_{\mathbf{1}}, \boldsymbol{u}_{\mathbf{2}}]$ is the 3×2 matrix whose columns are $\boldsymbol{u}_{\mathbf{1}}, \boldsymbol{u}_{\mathbf{2}}$.

Step 4: Projection to the ternary target space

Finally, to aid in the display and interpretation of the data, the datapoints that originally defined the three representative **5D** vectors $\boldsymbol{v}_{\mathbf{1}}, \boldsymbol{v}_{\mathbf{2}}, \boldsymbol{v}_{\mathbf{3}}$ from the dataset (substrate, streaky and spotty), in defined in 2D by the vectors $\boldsymbol{p}_{\boldsymbol{i}}$, are mapped to the vertices of an equilater target with side length 1, defined by the vectors $\boldsymbol{q}_{\boldsymbol{i}}$. This is done to obtain a pseudo-ternary plot of the growth RHEED patterns. First, the points $\boldsymbol{p}_{\mathbf{2}} - \boldsymbol{p}_{\mathbf{1}}$ and $\boldsymbol{p}_{\mathbf{3}} - \boldsymbol{p}_{\mathbf{1}}$ are mapped to $\boldsymbol{q}_{\mathbf{2}} - \boldsymbol{q}_{\mathbf{1}}$ and $\boldsymbol{q}_{\mathbf{3}} - \boldsymbol{q}_{\mathbf{1}}$, after which the point $\boldsymbol{p}_{\mathbf{1}}$ is translated by $\boldsymbol{b}$ such that it maps to $\boldsymbol{q}_{\mathbf{1}}$. The first mapping is achieved by computing $\boldsymbol{X''}_{\mathbf{2D}}\boldsymbol{A}^{\boldsymbol{T}}$, where $\boldsymbol{A} = \boldsymbol{N}\boldsymbol{M}^{-\mathbf{1}}$ and $\boldsymbol{M} = [\boldsymbol{p}_{\mathbf{2}} - \boldsymbol{p}_{\mathbf{1}},\ \boldsymbol{p}_{\mathbf{3}} - \boldsymbol{p}_{\mathbf{1}}]$ and $\boldsymbol{N} = [\boldsymbol{q}_{\mathbf{2}} - \boldsymbol{q}_{\mathbf{1}},\ \boldsymbol{q}_{\mathbf{3}} - \boldsymbol{q}_{\mathbf{1}}]$. The translation vector $\boldsymbol{b}$ is subtracted, such that $\boldsymbol{b} = \boldsymbol{q}_{\mathbf{1}} - \boldsymbol{A}\boldsymbol{p}_{\mathbf{1}}$. The complete affine transformation can then be written as:

$$\boldsymbol{X''}_{\boldsymbol{ternary}} = \left(\boldsymbol{X''}_{\mathbf{2D}}\boldsymbol{A}^{\boldsymbol{T}}\right) + \boldsymbol{b}.$$

Note S3: Dimensionality reduction pipeline

We applied a logarithmic transformation to each pixel to preprocess the 60x100 pixels RHEED images and reduce disparities between large and small pixel values. Subsequently, the images were standardized and normalized by dividing each by its maximum pixel value, ensuring consistent maximum intensity across all images. We first employed standard PCA to reduce the dataset's dimensionality from 6000 (flattened 60x100 images) to 500 components, retaining 98% of the variance. This reduction facilitated computational efficiency for the subsequent cPCA step (see Note S2), reducing dimensionality to 5 components.

The selection of the number of components N to retain and the value of the parameter α in cPCA was guided by a fitness function defined as:

$$\frac{\sum_{i=1}^{N} \lambda_i}{d_{RMS}/d_0},$$

where λ_i is the explained variance ratio corresponding to the i-th component, d_{RMS} the root mean square (RMS) distance from the centroid of the substrate's RHEED images before growth, computed in the N-dimensional space spanned by the first N cPCA components, and d_0 the RMS spread of the substrate's RHEED images before applying the cPCA. Using d_{RMS}/d_0 ensures that both the numerator and the denominator are elements of the [0,1] interval. λ_i is computed as the ratio of the variance of the i-th cPCA component and the total variance of the original dataset.

We computed the spread d_{RMS} and the cumulative explained variance $\sum_{i=1}^{N} \lambda_i$ for various α values and numbers of retained components N, as illustrated in Figure 1a-b. The heatmap of Figure 1c shows the computed fitness function, which reveals two maxima at distinct α values, at 36.9 and 428000. Figure 1d shows that while these two α regions exhibit significant differences in explained variance, the differences in RMS spread are minimal (note that d_0 for our dataset is 7). Considering this, and that large values of α favor the components with low background variance, but possibly penalizes components with interesting variations in the target dataset, we focused on the maxima at lower α values. At α=36.9 the explained cumulative variance curve starts flattening at 5 components, while the RMS spread does not show significant changes after 3 components. We therefore decided to retain 5 components, as the dataset is projected to 3D dimensional space in the next step.

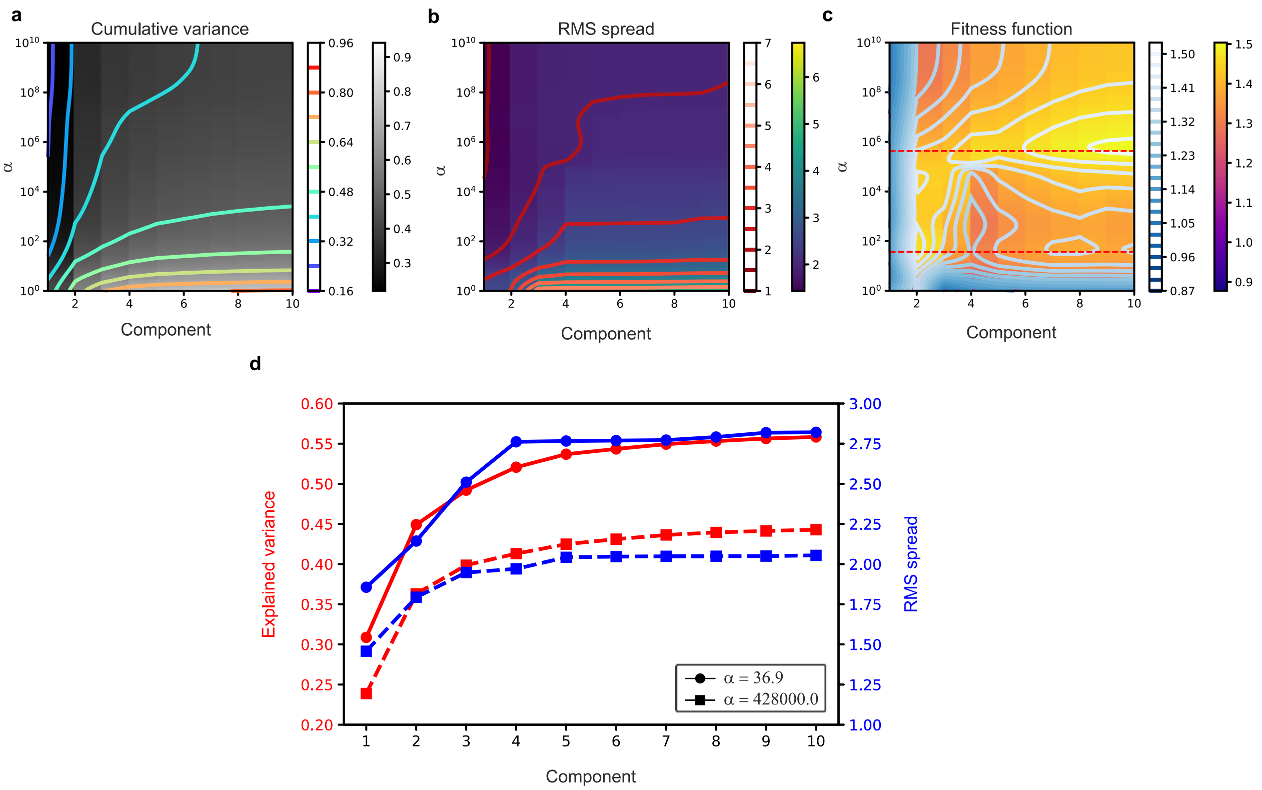

Figure S10: Variance and alpha. (a,b) Cumulative variance and N-dimensional RMS spread as a function of alpha and the number of retained components, respectively. **(c)** Heatmap of the fitness function indicating optimal alpha and component number pairs. **(d)** Values of explained variance and RMS spread corresponding to the maxima identified in (c) by the dashed lines.

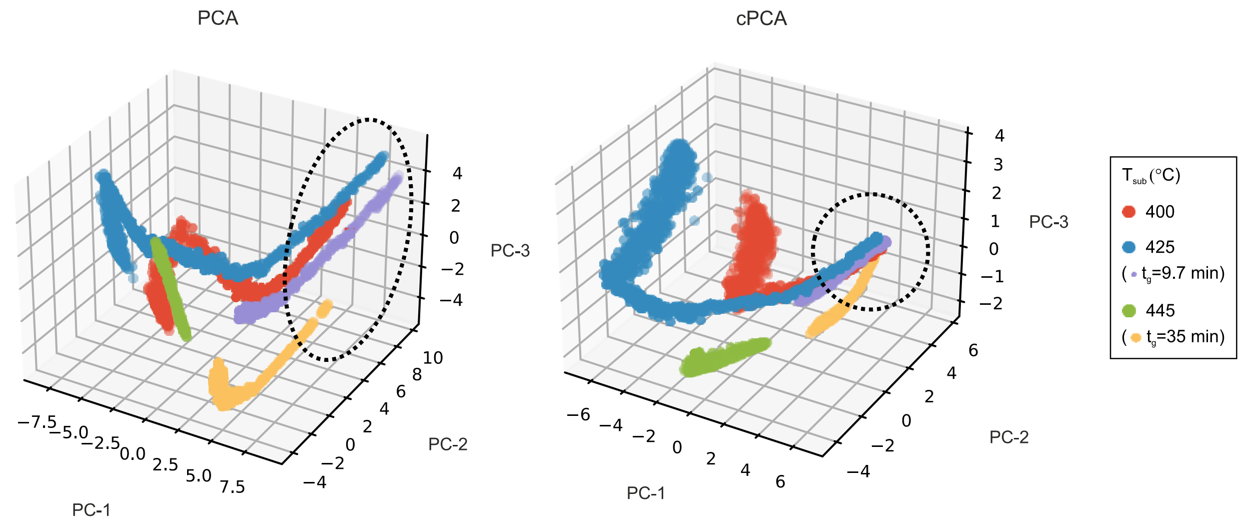

Figure S11: PCA and cPCA. Standard PCA (left) and cPCA (right) plotting only the first 3 components. It is important to note that cPCA identifies components that reduce background variance. This is shown by the substrate-related points closely in space unlike in standard PCA (see circled points).

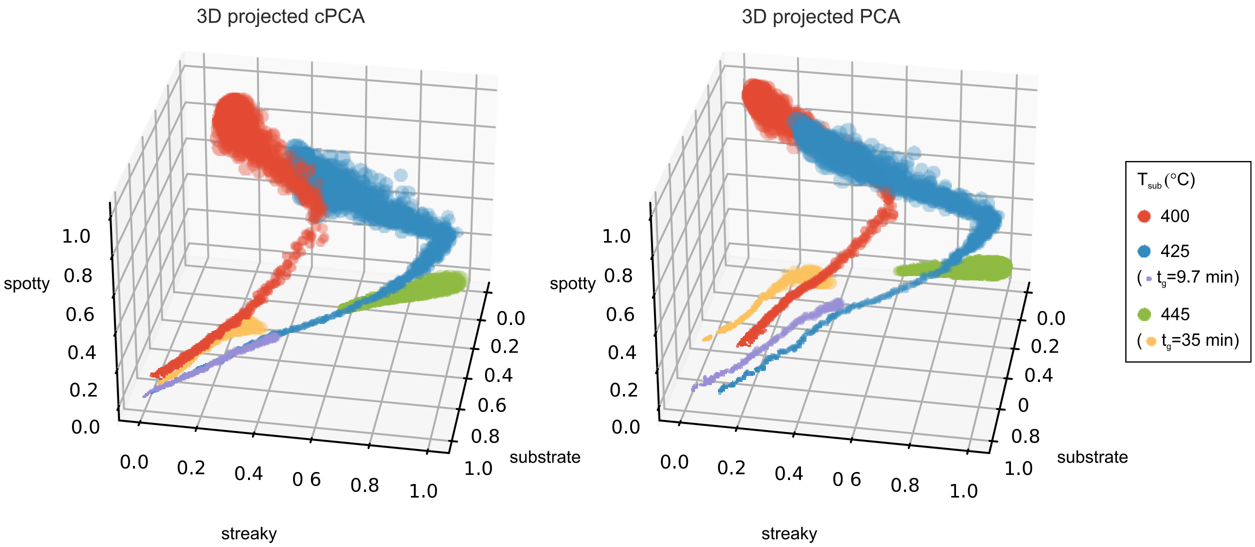

Figure S12: 3D projected cPCA and PCA. Projection of the 5D cPCA-analyzed time- and temperature-dependent RHEED data onto a 3D-dimensional space (spotty, streaky and substrate patterns). Dataset after the projection for the chosen α of 36.9 (left) and for α=0, which corresponds to standard PCA (right).

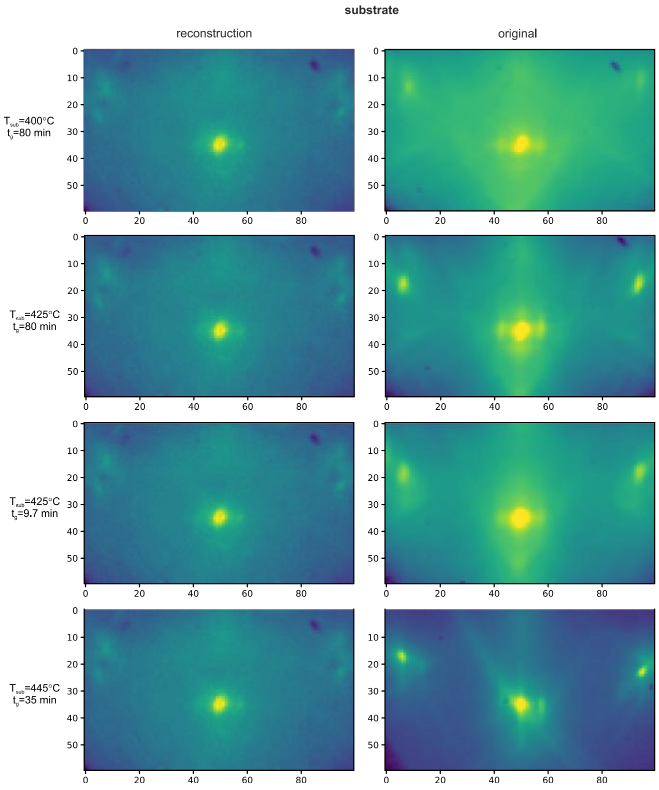

Figure S13: RHEED image reconstruction from 3D cPCA (start). Reconstructed RHEED images at the beginning of the growth (substrate) from the 2D highly reduced space obtained by the projection of the new 3D subspace back to the initial 6.000-dimensional space. The comparison with the original data shows how well the reduction preserves and captures the essential features.

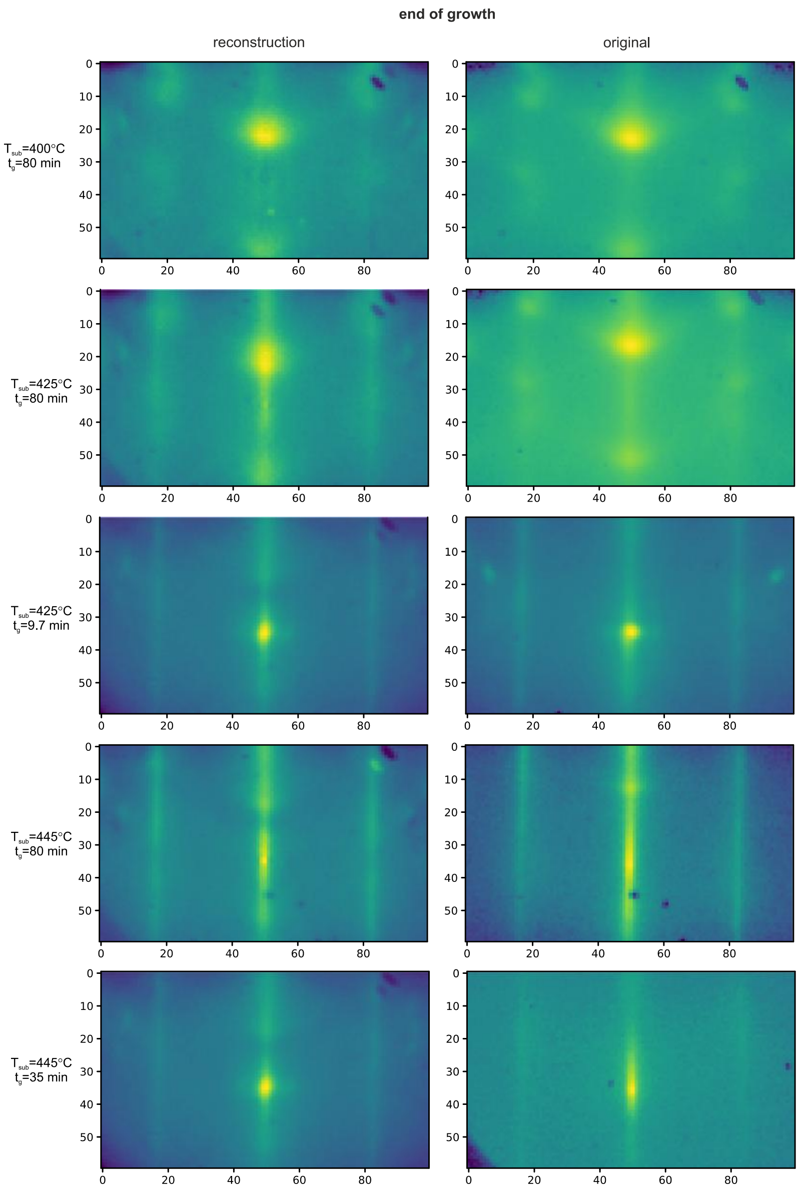

Figure S14: RHEED image reconstruction from 3D cPCA (end). Reconstructed RHEED images at the end of the growth from the 2D highly reduced space obtained by the projection of the new 3D subspace back to the initial 6000-dimensional space. The comparison with the original data shows the perfect match and that the reduction preserves and captures the essential feature.

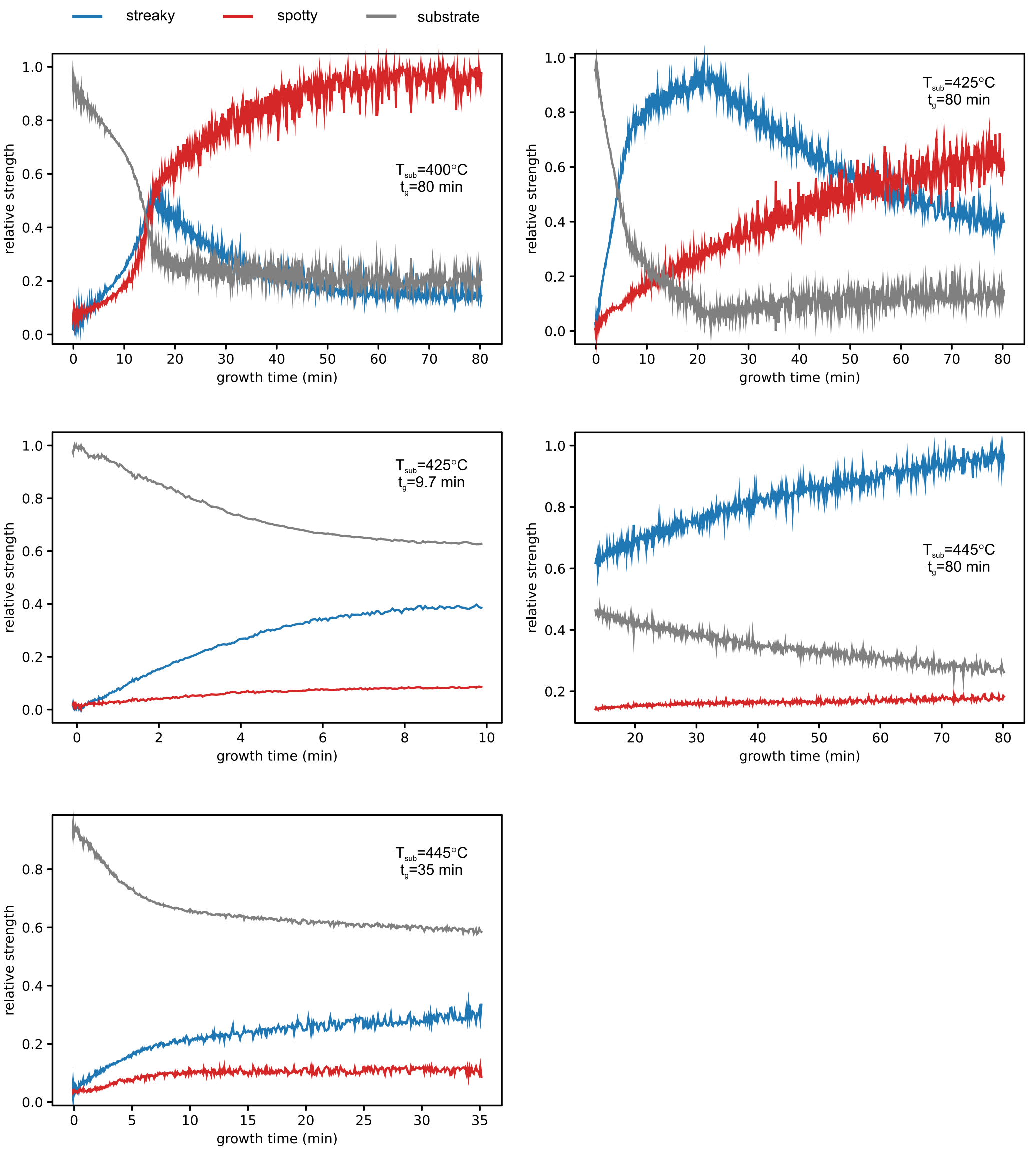

Figure S15: Growth trends. Percentages of substrate, spotty, and streaky patterns as a function of time for each growth run after normalization of the 5D data onto a 3D space. The observed trends perfectly reflect the growth modes described in the main text.

1. J Lopes, J.M., Czubak, D., Zallo, E., Figueroa, A.I., Guillemard, C., Valvidares, M., Rubio-Zuazo, J., López-Sanchéz, J., Valenzuela, S.O., Hanke, M., and Ramsteiner, M. (2021) Large-area van der Waals epitaxy and magnetic characterization of Fe_3GeTe_2 films on graphene. *2D Mater.*, **8** (4), 041001.
2. Cohen, P.I., Petrich, G.S., Pukite, P.R., Whaley, G.J., and Arrott, A.S. (1989) Birth-death models of epitaxy. *Surface Science*, **216** (1–2), 222–248.
3. Abid, A., Zhang, M.J., Bagaria, V.K., Zou, J. (2018) Exploring patterns enriched in a dataset with contrastive principal component analysis. *Nature Comm.* **9** (2134), 1.
